# Disruption of p62 ubiquitination by α-methylene-γ-butyrolactone enhances Keap1 lysosomal degradation for ulcerative colitis therapy

**DOI:** 10.1016/j.redox.2026.104424

**Published:** 2026-10-03

**Authors:** Ke-Gang Linghu, Dasong Wang, Tian Zhang, Fei Jiang, Long Wu, Di Pan, Yan Chen, Ling Tao, Ligen Lin, Hua Yu, Qianming Du, Weidong Pan, Xiangchun Shen

**Affiliations:** a State Key Laboratory of Discovery and Utilization of Functional Components in Traditional Chinese Medicine & School of Pharmaceutical Sciences, Guizhou Medical University, Guiyang, Guizhou, 561113, China; b Key Laboratory of Novel Anti-Cancer Drug Targets Discovery and Application, Guizhou International Science & Technology Cooperation Base for Druggability Research of Natural Medicines, Guizhou Medical University, Guiyang, Guizhou, 561113, China; c Department of Anus and Intestinal Surgery, The Affiliated Hospital of Guizhou Medical University, Guiyang, Guizhou, 550 025, China; d General Clinical Research Center, Nanjing First Hospital, Nanjing Medical University, Nanjing, 210006, China; e School of Basic Medicine & Clinical Pharmacy, China Pharmaceutical University, Nanjing, 210009, China; f State Key Laboratory of Mechanism and Quality of Chinese Medicine, University of Macau, Taipa, Macau, China

**Keywords:** Ulcerative colitis, α-Methylene-γ-butyrolactone, p62, Ubiquitination, Keap1, Autophagolysosome

## Abstract

Ulcerative colitis (UC) is an intestinal inflammatory disorder primarily driven by M1 macrophage activation and oxidative stress. p62/Sequestosome-1, a multifunctional autophagy adaptor protein, plays a critical role in regulating autophagy and inflammation. α-Methylene-γ-butyrolactone (αMγB), a conserved structural motif found in anti-inflammatory sesquiterpene lactones from traditional medicinal herbs, was shown to suppress M1 macrophage activation and inflammation. This study aimed to investigate the role of p62 in UC pathogenesis and evaluate the therapeutic potential of αMγB. We found that genetic deletion of p62 aggravated disease severity in both acute pneumonia and UC mouse models. Treatment with αMγB alleviated UC symptoms by reprogramming macrophage polarization, reshaping the mucosal immune microenvironment, and suppressing inflammatory crosstalk between macrophages and epithelial cells. Mechanistically, αMγB covalently modified p62 at C291, preventing its ubiquitination and stabilizing it, which enhanced Keap1 binding and drove its autolysosomal degradation. This process released Nrf2, facilitated its nuclear translocation, and activated the expression of downstream antioxidant and anti-inflammatory genes. Importantly, αMγB established a positive feedback loop between Nrf2 and p62 that sustained p62 upregulation and further promoted Keap1 autolysosomal degradation. These protective effects were abolished in p62 (C291S) mutant mice or upon Nrf2 inhibition. Collectively, our findings demonstrated that αMγB ameliorated UC by specifically targeting p62 at C291, promoting its stabilization, and enhancing Keap1 degradation, thereby modulating macrophage polarization. These findings highlighted the therapeutic potential of αMγB and suggested that augmenting p62 function might represent a promising strategy for UC treatment.

## Highlights

• p62 knockdown worsens acute pneumonia and experimental UC in mice.
• αMγB alleviates colitis by reprogramming macrophages and mucosal immunity.
• αMγB covalently targets p62-C291, reducing ubiquitination and stabilizing p62.
• p62 stabilization promotes autolysosomal Keap1 degradation and Nrf2 activation.
• p62-C291S mutation or Nrf2 inhibition abolishes αMγB efficacy in UC.

## Graphical abstract

**Figure:**
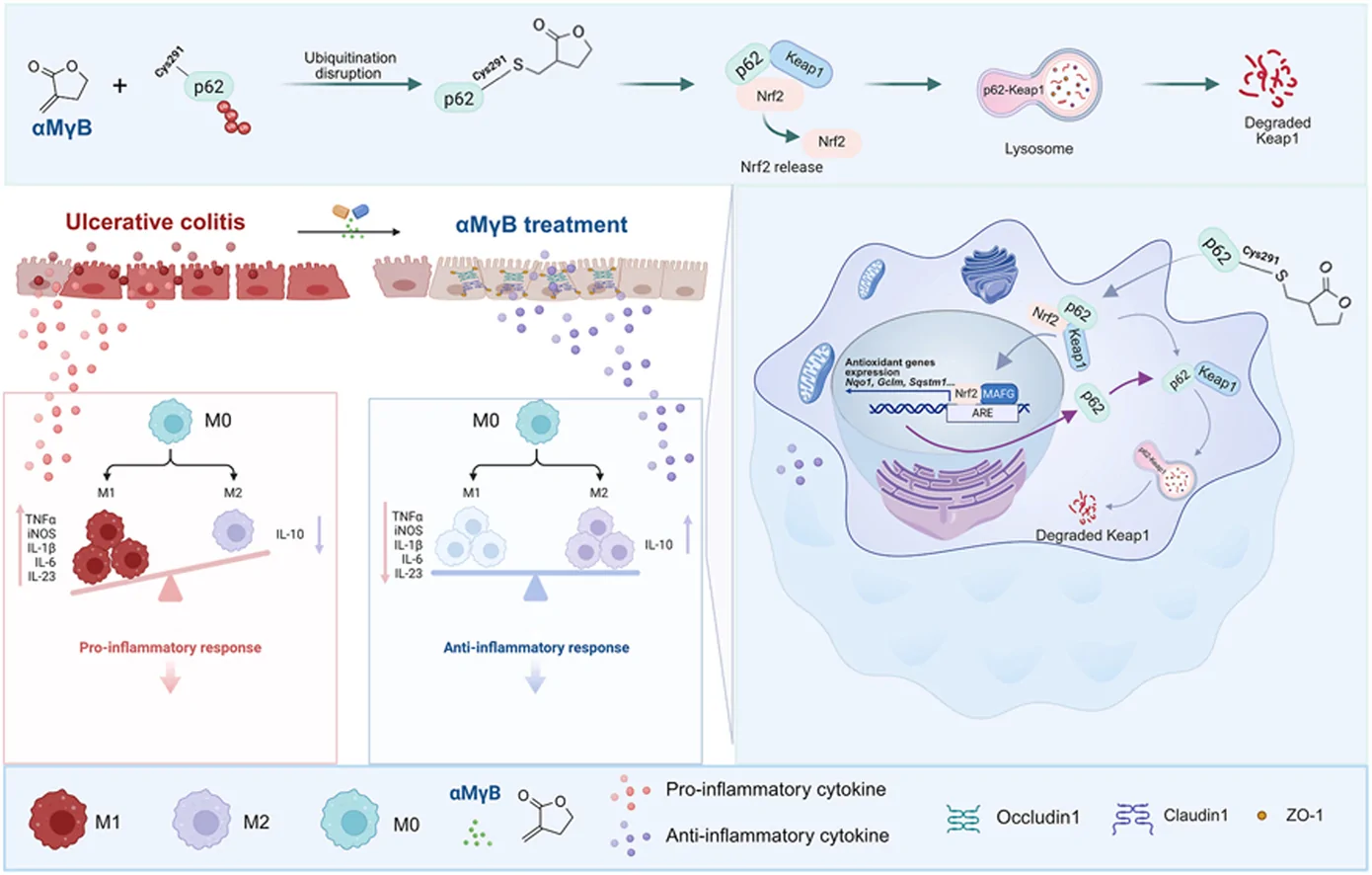

## 1 Introduction

Ulcerative colitis (UC) is a chronic inflammatory bowel disease characterized by recurrent intestinal inflammation, mucosal barrier dysfunction, and immune dysregulation. With its rising global incidence, UC significantly impairs quality of life and adds long-term risks, including the development of colorectal cancer, primary sclerosing cholangitis, and systemic complications related to chronic inflammation [1]. Although current therapies, such as aminosalicylates and biologics, have demonstrated considerable therapeutic efficacy, approximately 35% of patients exhibit inadequate responses due to primary nonresponse, loss of efficacy, or safety concerns [[2], [3], [4]]. Thus, there remains an urgent need to identify novel therapeutic targets and small-molecule agents for UC.

UC involves immune-oxidative colonic inflammation and a disrupted mucosal microenvironment (CMM) [5]. CMM integrity relies on macrophages for immune regulation and epithelial cells for barrier function; their dysfunction causes mucosal injury. Polarization of macrophages into pro-inflammatory M1 or anti-inflammatory M2 phenotypes critically influences disease progression. M1/M2 imbalance perpetuates inflammation and barrier damage via M1-derived ROS and cytokines (e.g., TNF-α, IL-6), which disrupt tight junctions, suppress goblet cells, and recruit neutrophils. Meanwhile, impaired M2 function further impedes healing [6,7]. Thus, promoting M1-to-M2 repolarization represents a promising therapeutic strategy for UC.

p62, a key adaptor in selective autophagy and UC pathogenesis, mediates degradation of ubiquitinated substrates [8]. Enhancing p62 function provides multi-mechanistic benefits: inhibiting NLRP3 inflammasome activation and NF-κB signaling, activating the Keap1-Nrf2 antioxidant pathway, and restoring intestinal barrier integrity [[9], [10], [11], [12], [13]]. Nuclear factor erythroid 2-related factor 2 (Nrf2), a central antioxidant transcription factor, ameliorates UC by orchestrating macrophage polarization [[14], [15], [16], [17]]. Nrf2 activity is regulated by Kelch-like ECH-associated protein 1 (Keap1). p62 binds Keap1 via its KIR domain, competitively disrupting Keap1-Nrf2 interaction and promoting Keap1 degradation, thereby activating Nrf2 and its binding to the ARE elements. p62 is itself transcriptionally regulated by Nrf2, forming the p62-Nrf2 positive feedback loop [[18], [19], [20]]. Targeting this circuit to modulate macrophage polarization and break the oxidative stress-inflammation cycle offers a promising therapeutic strategy for UC.

Natural sesquiterpene lactones (SLs), which are the active components of many traditional anti-inflammatory herbs such as *Sigesbeckia pubescens* Makino, demonstrate potent efficacy in inflammatory models. However, their development has been hindered by challenges such as complex purification procedures and limited natural abundance [[21], [22], [23], [24], [25], [26], [27], [28]]. In our previous studies, we designed α-methylene-γ-butyrolactone (αMγB), a simplified synthetic analog derived from the conserved structural motif of SLs. This compound preserves the evolutionarily conserved anti-inflammatory scaffold and effectively promotes macrophage repolarization from the pro-inflammatory M1 phenotype to the anti-inflammatory M2 phenotype. This compound demonstrates superior safety and robust anti-inflammatory efficacy both *in vitro* and *in vivo*, surpassing conventional agents such as aspirin, indomethacin, and dexamethasone [29,30]. The present study seeks to elucidate the molecular mechanisms by which αMγB inhibits M1 macrophage polarization in UC, thereby providing a mechanistic basis for the development of αMγB-derived therapeutics.

## 2 Materials and methods

### 2.1 Chemicals and reagents

Chemical and biological materials used in this study are listed in Supplementary Information Table S1.

### 2.2 Cell culture and differentiation

L929, RAW264.7, HEK293T, and NCM460 cells were cultured in DMEM medium supplemented with 10% fetal bovine serum (FBS), and 1% penicillin-streptomycin under a humidified atmosphere of 5% CO_2_ at 37 °C. THP-1 cells were maintained in RPMI-1640 medium and treated with PMA (100 ng/mL) for 12 h to induce differentiation into macrophages.

### 2.3 Isolation and culture of bone marrow-derived and peritoneal macrophages

Bone marrow-derived macrophages (BMDMs) and peritoneal macrophages (PEMs) were isolated from femurs/tibias and peritoneal cavities of 6-10-week-old male mice using established protocols [31]. Cells were cultured for 7 days in conditioned medium (10% FBS, 79% DMEM, 10% L929 supernatant, 1% penicillin-streptomycin) at 37 °C with 5% CO_2_ and 95% humidity.

### 2.4 Rationale for αMγB concentration and dose selection

The *in vitro* concentrations of αMγB (3-24 μM) were selected based on our previous cytotoxicity and anti-inflammatory activity data. αMγB showed no appreciable cytotoxicity at concentrations up to 100 μM and inhibited LPS-induced NO production with a half-maximal effective concentration (EC_50_) of approximately 8 μM. Accordingly, 3-24 μM was chosen to characterize its concentration-dependent activity. The *in vivo* doses of 2.5, 5, and 10 mg/kg were selected based on prior safety data and preliminary efficacy findings. The highest dose used (10 mg/kg) was one-tenth of the highest dose previously shown to be non-toxic (100 mg/kg/day, i.p., for 7 days), while preliminary experiments demonstrated marked, dose-dependent anti-inflammatory effects across the selected dose range [30].

### 2.5 Cell viability assay

The cell viability was determined using the MTT assay following the previously established experimental protocol [16]. Specifically, RAW264.7 and NCM460 cells were treated with specified concentrations of αMγB, and then incubated with MTT (1 mg/mL) for 4 h. Subsequently, the resulting formazan crystals were dissolved in DMSO, and absorbance was measured at 490 nm using a microplate reader.

### 2.6 Nitric oxide (NO) detection

The supernatants of RAW264.7 cells treated with LPS (200 ng/mL) and specified concentrations of αMγB for 12 h were collected. NO levels were quantified using the Griess reagent system according to the manufacturer's instructions and a previously established methodology [21].

### 2.7 Enzyme-linked immunosorbent assay (ELISA)

Cell culture supernatants, cell lysates, mouse serum, and tissue lysates were collected and analyzed using commercially available ELISA kits. All procedures were performed strictly following the manufacturer's instructions.

### 2.8 Macrophage-colonic epithelial cell Co-culture

Macrophage-normal colon epithelial cell co-culture was conducted following established protocols described in previous studies [32]. Effects of macrophages on NCM460 cells: THP-1 cells were differentiated into macrophages in the upper chambers using PMA (100 ng/mL), followed by 24 h culture in fresh medium. Concurrently, NCM460 cells were seeded in the lower chambers. Macrophages were then stimulated with LPS (100 ng/mL) and 24 μM αMγB for 12 h. After washing and medium replacement, macrophages in the upper chambers were co-cultured with NCM460 cells for 24 h, followed by the collection of supernatants and NCM460 cells. Effects of NCM460 cells on Macrophages: THP-1 cells were differentiated into macrophages in the lower chambers as above. NCM460 cells in the upper chambers were treated with TNF-α (20 ng/mL) and 24 μM αMγB for 24 h. After washing and medium replacement, NCM460 cells in the upper chambers were co-cultured with macrophages for 24 h, followed by the collection of supernatants and macrophages.

### 2.9 Cellular thermal shift assay (CETSA)

The drug-target engagement of αMγB was evaluated by CETSA following established protocols [30]. RAW264.7 cells were treated with or without 12 μM αMγB for 12 h. Cells were lysed and centrifuged (12,000 rpm, 20 min, 4 °C), then supernatants were adjusted to a 3 μg/μL protein concentration. Aliquots (60 μL) underwent thermal denaturation (37-95 °C, 3 min) followed by centrifugation. Target protein stability in soluble fractions was assessed by Western blotting.

### 2.10 Drug affinity responsive target stability (DARTS)

Target engagement was assessed using DARTS [33]. Cells were lysed and the lysates were clarified centrifugation. Then lysate aliquots were incubated with 12 μM αMγB or PBS control (37 °C, 1 h). Samples were divided into control group and digested with Pronase (10 mg/mL). αMγB- induced proteolytic resistance was analyzed by western blotting.

### 2.11 Label-free proteomic relative quantification analysis

RAW264.7 cells were treated with LPS (200 ng/mL) and/or αMγB (12 μM) for 12 h. After treatment, cells were washed twice with ice-cold PBS. Equal amounts of proteins were subjected to reduction and alkylation, followed by tryptic digestion with Trypsin. The digested peptides were analyzed by liquid chromatography-tandem mass spectrometry (LC-MS/MS). Raw mass spectrometry data were processed using MaxQuant software (version 1.6.2.10) for database searching, matching, and protein identification.

### 2.12 Transcriptome sequencing

BMDMs from C57BL/6J mice were cultured in 6-well plates to 80% confluency, treated with αMγB (6 μmol/L) for 6 h and lysed with TRIzol. The cellular lysates underwent high-throughput sequencing to capture all transcripts. The data were analyzed using the free online Majorbio Cloud Platform (www.majorbio.com).

### 2.13 Co-immunoprecipitation (Co-IP)

Cells treated with αMγB were lysed and centrifuged. Equal protein lysates were immunoprecipitated with target-specific antibodies overnight, followed by Protein A/G agarose bead incubation (40 μL, 4 h, 4 °C). Beads were washed and bound proteins eluted in SDS sample buffer (95 °C, 8 min) for western blotting analysis.

### 2.14 Assessment of autophagic flux

RAW264.7 cells were seeded on coverslips in 6-well plates at 5 × 10^4^ cells/well. After 12 h, cells were infected with GFP-mRFP LC3B lentivirus at the specified MOI for 24 h. Then cells were treated with LPS, αMγB, and CQ alone or in combination for 12 h. Following PBS washes, cells were fixed in 4% PFA for 20 min and imaged by confocal microscopy.

### 2.15 Establishment of an acute inflammation mouse model

C57BL/6J mice (both wild-type and *Sqstm1*^*−/−*^ knockout) were randomly divided into two groups: control and LPS-model groups. Control mice received intraperitoneal saline injections, while model groups received LPS (2 mg/kg) intraperitoneally. All samples were collected 4 h post-injection.

### 2.16 Establishment of ulcerative colitis mouse model and treatment

C57BL/6J mice (wild-type, *Sqstm1*^*−/−*^ knockout, adeno-associated virus (AAV9)-*Sqstm1*, and AAV9-*Sqstm1* (C291S)) were randomly divided into three groups: control group, Dextran Sulfate Sodium Salt (DSS) group, and drug-treated groups. Control group received normal drinking water for 14 days. DSS model group were administered 3% DSS solution ad libitum for 7 days, followed by normal water for 7 days. Drug-treated groups received daily intraperitoneal injections of the indicated compounds for 14 days. During modeling, mice were weighed daily and evaluated for bloody stools/stool consistency to calculate the disease activity index (DAI) using established criteria [34]. On day 15, mice were euthanized under anesthesia. In parallel, C57BL/6J mice were assigned to control and αMγB groups and received daily intraperitoneal injections of PBS or αMγB (10 mg/kg) for 14 days without DSS treatment. On day 15, the heart, liver, spleen, lung, and kidney were collected for histopathological evaluation.

### 2.17 Transgenic mice establishment

Adult male wild-type C57BL/6J mice, aged 7 to 8 weeks, were procured from Guizhou Laboratory Animal Engineering Technology Center, Guizhou Medical University. Adult male C57BL/6J-*Sqstm1*^*+/−*^ knockout mice were procured from Saiye (Suzhou) Biotechnology Co., Ltd. These mice were bred with wild-type (WT) female adults to generate C57BL/6J-*Sqstm1*^*+/−*^ heterozygotes or WT mice. Genomic DNA was extracted from mouse tail samples using the TaKaRa MiniBEST Universal Genomic DNA Extraction kit (Ver.5. Code No. 9765) as per the manufacturer's instructions. PCR amplification was performed using primer set 1, and the resulting products were analyzed by agarose gel electrophoresis for genotyping. A 431 bp fragment confirmed successful deletion of the target gene in primer set 1, while the WT fragment was 7470 bp. Subsequently, heterozygous adult males and females were bred to generate C57BL/6J-*Sqstm1*^*−/−*^ male homozygotes. PCR amplification was carried out using specific primer sets, and genotyping was determined. The genotype-specific primers for identification are listed in the Supplementary Information Table S3.

AAV9-*Sqstm1*, AAV9-*Sqstm1* (C291S), and AAV9-ZsGreen adeno-associated viral vectors were purchased from Hanheng Biotechnology Co., Ltd. (Shanghai, China). The above three adeno-associated viruses were delivered into the colon of adult male C57BL/6J-*Sqstm1*^*−/−*^ homozygous mice via intracolonic administration to restore wild-type *Sqstm1* expression or express the C291S mutant in *Sqstm1*^*−/−*^ mice. Stable viral expression in the colon was confirmed for 4 weeks post-injection.

All mice were maintained under specific pathogen-free (SPF) conditions with a temperature of 22 ± 2 °C, humidity of 50 ± 5%, and a 12/12 h light/dark cycle. All experimental protocols involving animals received approval from the experimental animal ethics committee at Guizhou Medical University (Approval No. 2400326) and strictly adhered to established guidelines and regulations.

### 2.18 Tissue samples from colon cancer patients

Paired human colorectal cancer (CRC) tissues and their corresponding adjacent non-tumor tissues (n = 5) were obtained from patients undergoing surgical resection at the Affiliated Hospital of Guizhou Medical University. The study protocol was approved by the Ethics Committee of the Affiliated Hospital of Guizhou Medical University (Approval No.: 2025211), and written informed consent was obtained from all participants prior to enrollment. Following surgical excision, all tissue specimens were immediately snap-frozen in liquid nitrogen and stored at -80 °C for subsequent RT-qPCR and Western blot analysis.

### 2.19 Immunofluorescence (IF)

RAW264.7 cells in confocal dishes were fixed with 4% PFA. After incubation with primary antibodies and fluorophore-conjugated secondary antibodies (1 h), nuclei were counterstained with DAPI (1 μg/mL, 5 min). Images were acquired using a confocal microscope.

Deparaffinized/rehydrated colon sections were blocked with 5% goat serum (30 min). Sections were incubated with primary antibodies and fluorescent secondary antibodies (2 h), followed by DAPI nuclear staining (1 μg/mL, 5 min). Protein localization was analyzed by fluorescence microscopy.

### 2.20 Histological analysis

Colon tissues were fixed in 4% PFA, paraffin-embedded, and sectioned (5 μm). Following deparaffinization/rehydration, sections underwent Hematoxylin and Eosin (H&E) staining per standard protocols to evaluate histopathology [35].

### 2.21 Immunohistochemistry (IHC)

Paraffin-embedded colon sections (5 μm) were deparaffinized in xylene and rehydrated through a graded ethanol series. Antigen retrieval was performed in 0.01 M citrate buffer (pH 6.0) in a boiling-water bath for 2 min. Endogenous peroxidase activity was quenched with 3% H_2_O_2_ for 10 min at room temperature, and nonspecific binding was blocked with 5% normal goat serum for 30 min. Sections were incubated overnight at 4 °C with primary antibodies against ZO-1, Occludin 1, IL-1β, p62, Nrf2, CD86, or CD206; detailed antibody information is provided in Table S1. After PBS washing, the sections were incubated with the appropriate HRP-conjugated secondary antibodies for 2 h at room temperature. Immunoreactivity was visualized using DAB, and the nuclei were counterstained with hematoxylin before imaging by light microscopy.

### 2.22 Real-time reverse transcription quantitative PCR (RT-qPCR)

Total RNA was isolated using TRIzol reagent. 1 μg RNA was reverse-transcribed into cDNA using Hifair® V RT SuperMix. Quantitative PCR amplification was performed with Hieff UNICON® SYBR Green Master Mix on a CFX Connect™ Thermal Cycler using gene-specific primers (Supplementary Information Table S2), per manufacturer's protocols.

### 2.23 Nrf2-ARE binding assay

Nrf2-ARE binding activity was evaluated using an ARE Reporter Kit (Catalog #60514, BioScience Inc.) according to established protocols [16]. HEK293T cells were seeded into 96-well cell culture plates at a density of 6 × 10^3^ cells per well. The cells were then transfected for 24 h with either 1 μL of the ARE reporter gene (60 ng/μL; containing both ARE-driven firefly luciferase reporter and constitutively expressed Renilla luciferase vectors) or the negative control gene (non-inducible firefly luciferase reporter with constitutively expressed Renilla luciferase vector). Cells were then treated with specified αMγB concentrations for 24 h. Nrf2-ARE binding activity was quantified using the Dual-Luciferase Reporter Assay System (Promega #E1910).

### 2.24 Western blotting analysis

Protein lysates from cells/tissues were extracted using RIPA buffer. Supernatant concentrations were quantified by BCA assay and adjusted to 2-4 μg/μL. Proteins underwent SDS-PAGE separation followed by PVDF membrane transfer. Membranes were blocked with 5% BSA and incubated with primary antibodies (overnight, 4 °C). After washing, secondary antibody incubation preceded ECL-based detection. Protein bands were imaged (Bio-Rad gel documentation system) and quantified (Image-Lab software).

### 2.25 Covalent molecular docking

Molecular docking was performed in Schrödinger 2019-01. The αMγB compound was modeled in ChemDraw, energy-minimized (Chem3D), and processed through Maestro's LigPrep module for hydrogen addition, optimization, and minimization. The SQSTM1 structure (UniProt: Q64337) was prepared via Protein Preparation Wizard with hydrogen addition, geometric refinement, and OPLS3e minimization. Covalent docking (Glide) employed a 10 × 10 × 10 Å grid centered on catalytic cysteines (C26, C113, C219, C291).

### 2.26 Plasmid amplification, extraction, and transfection

The mouse siRNA targeting *Sqstm1* (si-*Sqstm1*) was purchased from Sangon Biotech (Shanghai, China). Plasmids encoding p62-WT and its cysteine mutants (C26S, C113S, C219S, C291S) were obtained from Aiji Biotechnology Co., Ltd. (Guangzhou, China). Plasmids were amplified in liquid culture medium supplemented with appropriate antibiotics. Plasmid DNA was extracted using the SPARKeasy Endofree Midi Plasmid Kit according to the manufacturer's instructions. DNA concentration and purity were measured using a NanoPhotometer N60 Touch (IMPLEN, Germany). RAW264.7 cells were transfected with plasmids or siRNA using Liposome Transfection Reagent 3000, following the reagent protocol.

### 2.27 Oxidative stress assessment

Measurement of intracellular ROS. RAW264.7 cells cultured in 6-well plates for 12 h were treated for 12 h with LPS, αMγB, ML385, NAC, or si-*Sqstm1*, either individually or in combination. Cells were subsequently stained with 10 μM DCFH-DA for 30 min in the dark, washed with PBS, and intracellular fluorescence intensity was quantified by flow cytometry (Agilent Technologies, CA, USA).

Measurement of oxidative stress levels in mouse serum and colonic tissues. Oxidative stress markers (MDA, SOD, GSH, CAT) in mouse serum and colonic tissues were quantified using commercial assay kits (Abbkine Scientific Co., Ltd.) according to manufacturers’ instructions. Tissue homogenates and serum samples were prepared by centrifugation, with results normalized to total protein concentration or serum volume.

### 2.28 Flow cytometric analysis of T cell subsets and macrophage polarization

Splenic CD3e^+^CD4^+^CD8α^+^ T cell subsets were analyzed as described [36]. Single-cell suspensions from the mouse spleen, mesentery, and colonic lamina propria (cLP) were prepared for flow cytometric analysis. For the spleen, tissues were dissociated through a 200-μm nylon mesh and centrifuged (1500 rpm, 5 min). Erythrocytes were removed using red blood cell lysis buffer (Solarbio, China).

For the mesentery and colonic lamina propria, tissues were minced and pretreated with EDTA to remove epithelial cells, then enzymatically digested with Collagenase III (37 °C, 30 min). The resulting digests were filtered through a 70-μm cell strainer and centrifuged. After erythrocyte lysis and resuspension in PBS, cell counts were performed.

To analyze CD3e^+^CD4^+^CD8α^+^ T cell subsets, cells were stained with a fluorophore-conjugated antibody cocktail containing anti-CD3e, anti-CD4, and anti-CD8α for 30 min at 4 °C in the dark. To evaluate macrophage polarization in the colonic lamina propria, cells were labeled with antibodies against CD86 (M1 marker) and CD206 (M2 marker) following initial gating for pan-macrophages (e.g., F4/80 or CD11b). After PBS washing, data were acquired with a flow cytometer and analyzed with FlowJo software.

### 2.29 Synthesis of αMγB-Linker, click chemistry and pull-down assay

The synthesis of αMγB-Linker, click chemistry, and pull-down assay were strictly conducted following the previously established methods [16,37]. Proteins were extracted from RAW264.7 cells treated with αMγB-Linker or αMγB using RIPA lysis buffer. For click chemistry conjugation, 900 μL of lysate was incubated with a reaction premix containing 2 μL of 100 mM TBTA, 2 μL of 1 M CuSO_4_, 2 μL of 1 M TCEP, and 5 μL of 20 mM biotin-PEG_3_-azide at 37 °C for 2 h under light-protected conditions with vigorous shaking. Proteins were precipitated by adding 8 vol of ice-cold methanol and incubating at −20 °C for ≥30 min. After centrifugation, the pellet was washed with 5 mL methanol and air-dried. The supernatant was incubated with 50 μL of Pierce™ High Capacity NeutrAvidin™ Agarose beads overnight at 4 °C on a rotator, followed by 1 h at RT. Beads were washed and bound proteins were eluted in sample loading buffer and analyzed via western blotting.

### 2.30 Data analysis

Data are presented as the mean ± SD and were derived from at least three independent experiments. All data were assessed for normality using the Shapiro-Wilk test. Normally distributed data were analyzed using the parametric Student's t-test; otherwise, the nonparametric Mann-Whitney test was used. For comparisons involving more than two groups, one-way ANOVA was used for normally distributed data, whereas the Kruskal-Wallis test was used for non-normally distributed data. All n values reported in the manuscript represent biological replicates. *P* < 0.05 was considered statistically significant, and ns indicates no significant difference. Statistical analyses were performed using GraphPad Prism 9.5.1.

## 3 Results

### 3.1 p62 knockdown mice exhibit an exacerbated inflammatory phenotype

Patients with UC exhibit a significantly elevated risk of colorectal cancer (CRC) due to recurrent mucosal damage from chronic inflammation [2,38]. These observations suggest that patients with CRC and UC may share certain pathological features. GEPIA2 database analysis revealed a slight upregulation of *SQSTM1* expression in CRC tissues (Fig. 1A), which was further confirmed in clinical CRC samples and colorectal cancer cell lines (Fig. 1B–D; Fig. S1A–C). Based on this observation, we hypothesized that p62 may act as a pathological factor involved in the progression from UC to CRC. To elucidate the role of p62 in UC and inflammatory pathology, we generated p62-knockdown mice, and established LPS-induced acute pneumonia and DSS-induced UC models (Fig. 1E).

**Fig. 1:**
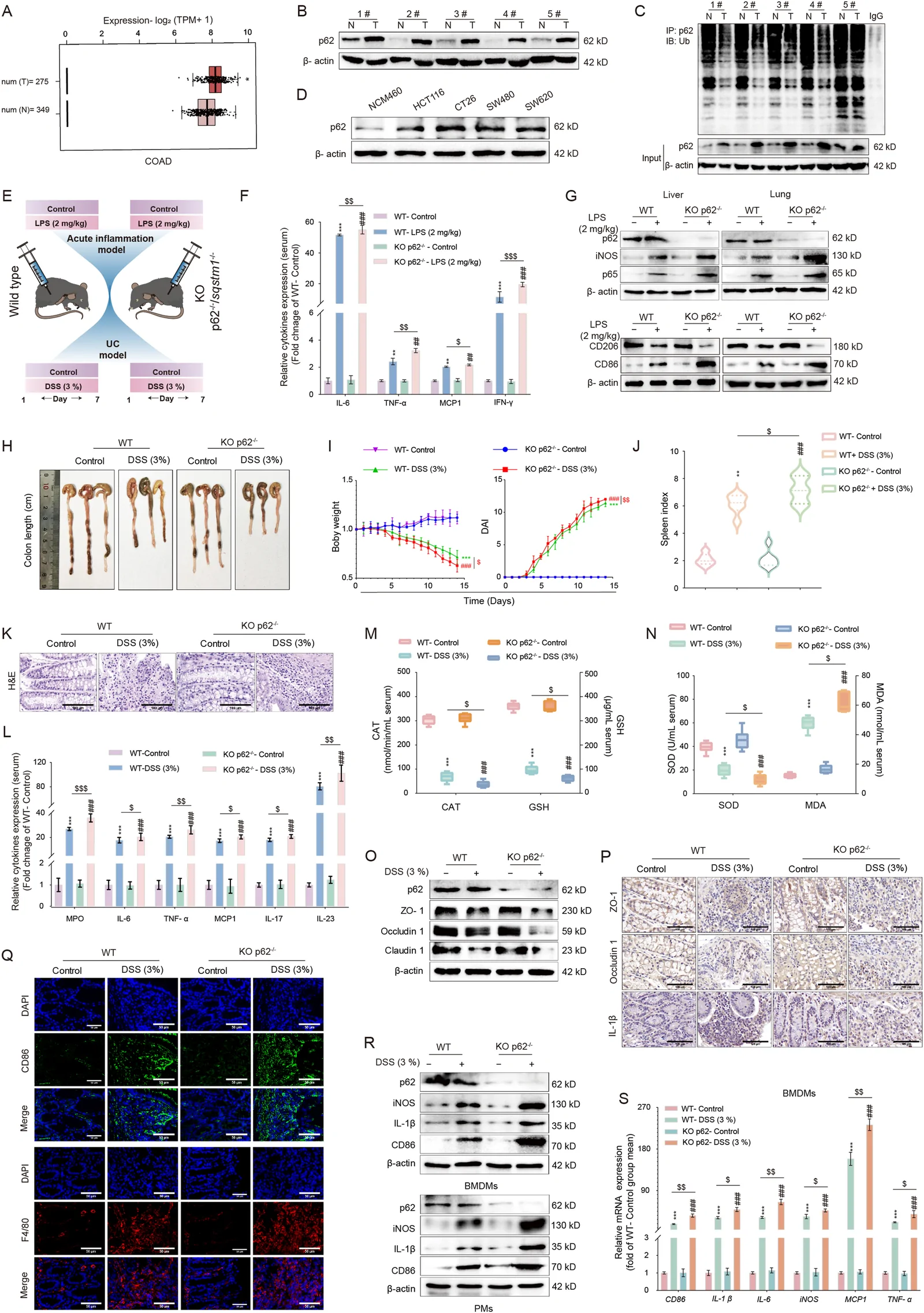
**p62 knockdown mice exhibit an exacerbated inflammatory phenotype.** (A) GEPIA2 analysis showed an upregulation of *SQSTM1* mRNA in colon cancer patients (N: normal; T:tumor). (B-C) We observed increased p62 protein expression alongside reduced ubiquitination of p62 in colon cancer tissues (N: normal; T:tumor; n pairs = 5). (D) Compared to normal colonic epithelial cells (NCM460), p62 protein expression was upregulated in colon cancer cells (HCT116, SW620, SW480 and CT26) (n = 3). (E) Acute inflammation and UC models were established using wild-type and p62-knockout mice, respectively. (F) Inflammatory cytokine levels in serum from acute inflammation mouse models (n = 5). (G) Expression levels of p62, p65, and iNOS proteins in murine liver and lung tissues (n = 5; iNOS and p65 are core inflammatory markers. p62 promotes Keap1 degradation, thereby activating the Nrf2 pathway, which subsequently antagonizes NF-κB (p65) signaling by inhibiting its nuclear recruitment and DNA-binding activity. This suppression leads to decreased iNOS transcription, ultimately mitigating oxidative stress and inflammatory injury). (H) Colon length in mice (n = 5). (I) Body weight changes and DAI scores in mice (n = 5). (J) Spleen index in mice (n = 5). (K) Histological analysis of colon tissue sections (H&E staining) (Scale bar = 100 μm; n = 5). (L) Quantification of inflammatory cytokines in serum from UC mouse models (n = 5). (M − N) Oxidative stress markers (SOD, GSH, CAT, MDA) in serum from UC mouse models (n = 5). (O-P) Reduced colonic tight junction proteins (Occludin 1, Claudin 1, ZO-1) and elevated pro-inflammatory IL-1β in p62-knockout UC mice (scale bar = 100 μm; n = 5). (Q-S) M1 macrophage polarization was exacerbated in p62-knockout UC mice (Colon tissues, BMDMs, PMs; scale bar = 50 μm; markers: IL-6, IL-1β, TNF-α, MCP-1, CD86, iNOS; n = 5). *P < 0.05, **P < 0.01 and ***P < 0.001 vs. the WT- Control, while ^*#*^*P* < 0.05, ^*##*^*P* < 0.01 and ^*###*^*P* < 0.001 vs. the KO p62^−/−^- Control, while ^*$*^*P* < 0.05, ^*$$*^*P* < 0.01 and ^*$$$*^*P* < 0.001 vs. the WT- LPS and WT- DSS (3%).

Contrary to our initial hypothesis, in p62-knockdown mice with acute inflammation, the protein and mRNA levels of pro-inflammatory mediators (IL-6, TNF-α, MCP1, IFN-γ, p65 and iNOS) were markedly increased in serum, liver, and lung tissues compared to wild-type mice with acute inflammation (Fig. 1F–G; Fig. S1D–G). Moreover, the M1 macrophage polarization was significantly exacerbated following p62 knockdown (Fig. 1G; Fig. S1H–J). Similarly, p62 knockdown exacerbated DSS-induced pathological changes, including colon shortening, weight loss, elevated spleen index, increased disease activity index (DAI), and impaired colon structural integrity (Fig. 1H–K; Fig. S1K–L). Furthermore, p62 knockdown led to elevated levels of pro-inflammatory cytokines (IL-6, IL-1β, TNF-α, MCP-1, IL-17, IL-23) and Myeloperoxidase (MPO) in both serum and colon tissues (Fig. 1L; Fig. S1M).

M1 macrophage-derived TNF-α disrupts intestinal tight junction proteins (Occludin 1, Claudin 1, ZO-1) and induces apoptosis, while IL-1β/IL-6 amplify inflammation, and ROS/NO-mediated oxidative damage synergistically impairs barrier integrity, promoting bacterial translocation [[39], [40], [41]]. Consistent with this observation, p62-knockdown UC mice exhibited a spectrum of aggravated pathologies, including exacerbated oxidative damage, loss of tight junction proteins (ZO-1, Occludin 1, Claudin 1), enhanced macrophage infiltration into the colon, and heightened M1 polarization (Fig. 1M–S; Fig. S1N–O; Fig. S2A–D). Collectively, the above data underscore the critical role of p62 expression against UC and related inflammatory conditions.

### 3.2 p62/Nrf2 enhancement by the natural product-derived activator attenuates UC

α-Methylene-γ-butyrolactone (αMγB) represents a conserved structural motif present in anti-inflammatory sesquiterpene lactones derived from traditional medicinal herbs. As we previously reported, αMγB inhibited the NF-κB signaling pathway and attenuated inflammation and oxidative stress by repolarizing M1 macrophages to the M2 phenotype [29,30]. To explore the exact anti-UC mechanisms, we used label-free quantitative proteomics and found that αMγB significantly upregulated p62 protein expression in macrophages, a finding further validated by complementary assays (Fig. 2A–C; Table S4; Fig. S3A–B). Furthermore, p62 knockdown in macrophages led to a loss of the ability of αMγB to regulate macrophage polarization (Fig. 2D–E; Fig. S3C–F). Transcriptome sequencing was performed on mouse bone marrow-derived macrophages (BMDMs) treated with αMγB in the presence or absence of LPS. Differentially expressed genes (DEGs) were subsequently identified, and KEGG enrichment analysis further revealed that these DEGs were significantly associated with the inflammatory bowel disease (IBD) pathway (Fig. 2F). To this end, we investigated the role of αMγB in colonic epithelial cells. We found that αMγB not only upregulated p62 expression in NCM460 cells but also protected these cells against TNF-α-induced inflammatory injury by suppressing pro-inflammatory mediators (e.g., IL-1β, IL-6, iNOS) and enhancing anti-inflammatory IL-10 expression (Fig. 2G–H; Fig. S3G–I). These findings align with observations in LPS-stimulated RAW264.7 macrophages. Furthermore, αMγB inhibited the adhesion of THP-1 monocytes to NCM460 cells in monocyte adhesion assays (Fig. 2I; Fig. S3J), a process relevant to early UC pathology, in which circulating monocytes infiltrate the mucosa and differentiate into macrophages.

**Fig. 2:**
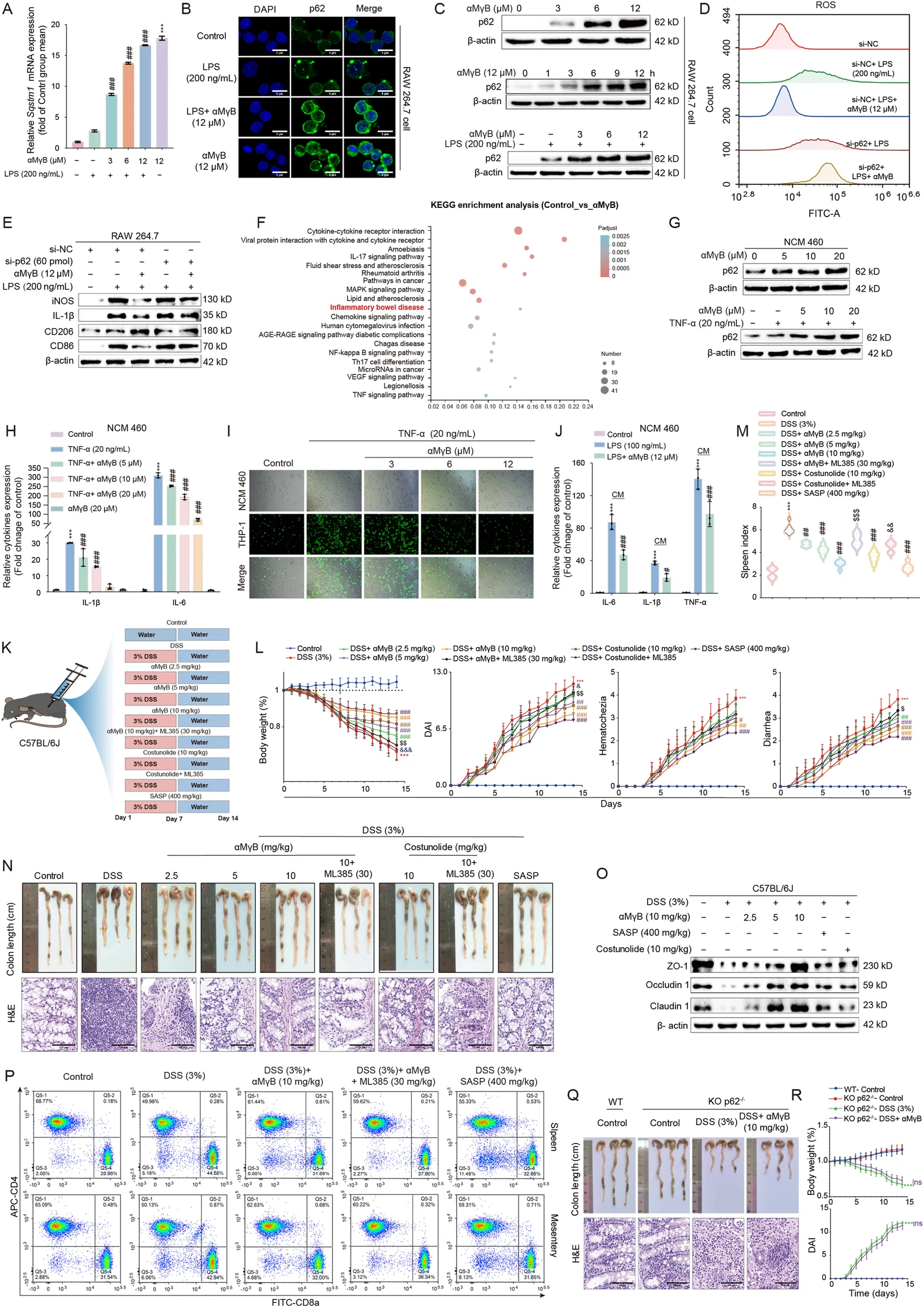
**p62 enhancement by the natural product-derived activator attenuates UC.** (A) αMγB significantly increases *Sqstm1* mRNA levels (n = 3). (B-C) αMγB promotes p62 protein expression in a time- and dose-dependent manner (Scale bar = 5 μm; n = 3). (D) p62 silencing reverses αMγB's suppression of ROS (n = 3). (E) p62 silencing reverses αMγB's suppression of M1 markers (CD86, iNOS, IL-1β) and induction of M2 markers (CD206) (n = 3). (F) KEGG enrichment analysis (bubble plot). (G) αMγB dose-dependently increases p62 protein in ±TNF-α-treated NCM460 cells (n = 3). (H) Inflammatory cytokine levels in NCM460 cells supernatants (n = 3). (I) αMγB inhibits THP-1 monocyte (pre-labeled with BCECF/AM) adhesion to NCM460 colonic epithelial cells (original magnification×20; n = 3). (J) In macrophage-colon epithelial co-culture systems, αMγB blocks inflammatory crosstalk between M1 macrophages and TNF-α-stimulated epithelial cells (n = 3). (K) DSS-induced UC model establishment. (L) Disease progression metrics: body weight change, diarrhea score, fecal bleeding score, and Disease Activity Index (DAI) (n = 5). (M) Splenic index (spleen weight/body weight ratio; n = 5). (N) Representative colon lengths and H&E staining showing colon tissue pathology (scale bar = 100 μm; n = 5). (O) αMγB restores expression of tight junction proteins (ZO-1, Occludin 1, Claudin 1) in colon tissue of UC mice (n = 5). (P) αMγB restores CD4^+^/CD8^+^ T-cell ratios in splenic and colonic mucosal tissues (n = 5). (Q) Representative colon lengths and H&E staining showing colon tissue pathology (scale bar = 100 μm; n = 5). (R) Body weight and DAI of mice (n = 5). *P < 0.05, **P < 0.01 and ***P < 0.001 vs. the Control, the si-NC, while ^*#*^*P* < 0.05, ^*##*^*P* < 0.01 and ^*###*^*P* < 0.001 vs. the si-NC + LPS (200 ng/mL), the TNF-α (20 ng/mL) and the DSS (3%), while ^*$*^*P* < 0.05, ^*$$*^*P* < 0.01 and ^*$$$*^*P* < 0.001 vs. the si-NC + LPS+αMγB, the DSS+ αMγB, while ^*&*^*P* < 0.05, ^*&&*^*P* < 0.01 and ^*&&&*^*P* < 0.001 vs. the Costunolide.

Subsequently, we established a Transwell co-culture system. Conditioned medium (CM) from LPS-stimulated macrophages significantly increased the release and mRNA levels of pro-inflammatory cytokines (IL-1β, IL-6, TNF-α) in colonic epithelial cells, along with elevated *MCP-1* and *IFN-γ* mRNA. In contrast, CM from macrophages treated with LPS plus αMγB reversed these pro-inflammatory effects and increased *IL-*10 mRNA in colonic epithelial cells (Fig. 2J; Fig. S3K). Similarly, CM from αMγB-treated colonic epithelial cells suppressed macrophage activation and enhanced the IL-10 expression (Fig. S3L–M), suggesting the existence of a cycling loop formed by pathogenic macrophages and colonic epithelial cells, while αMγB bidirectionally abrogates this loop. Collectively, these results underscore that the regulation of crosstalk between macrophages and colonic epithelial cells is the key mechanism underlying the therapeutic efficacy of αMγB in UC.

Thereafter, a mouse UC model was established to validate the therapeutic effect of αMγB on IBD (ML385, a specific Nrf2 inhibitor, functions by impairing Nrf2 post-nuclear stability and transcriptional activity; sulfasalazine (SASP) was used as a positive control drug in this study to evaluate the anti-UC activity of αMγB) (Fig. 2K). As expected, αMγB significantly alleviated typical symptoms in UC mice, including weight loss, bloody diarrhea, elevated spleen index, colon shortening, and inflammatory infiltration (Fig. 2L–N; Fig. S3N–O). It restored tight junction protein expression and reduced oxidative damage, thereby enhancing mucosal barrier integrity and limiting pathogen penetration (Fig. 2O; Fig. S3P–Z; Fig. S4A–B). Treatment with αMγB also downregulated pro-inflammatory mediators (IL-6, IL-1β, TNF-α, MCP-1, IL-17, IL-23, MPO) and M1 polarization markers, while increased M2 markers and IL-10 levels of serum and tissues (Fig. S4 C-L; Fig. S5A–B). Flow cytometry further indicated that αMγB ameliorated DSS-induced immune deficiency by elevating CD4^+^/CD8α^+^ T cell ratio in the spleen and mesentery (Fig. 2P; Fig. S5C–D). Furthermore, αMγB induced a reconfiguration of the gut microbiota, characterized by altered α- and β-diversity and an expansion of beneficial bacteria (Fig. S5E–I). More importantly, in line with the *in vitro* results, treatment with αMγB led to a significant upregulation of p62 protein in the colon tissues of mice with UC (Fig. S5J–M). Moreover, the protective effect of αMγB on UC was attenuated after p62 knockdown (Fig. 2Q–R; Fig. S5N–R), thereby confirming the consistency between *in vivo* and *in vitro* results.

The αMγB dose used here (10 mg/kg) was tenfold lower than a previously tested non-toxic dose (100 mg/kg/day, i.p., for 7 days), which caused no changes in liver enzymes or liver, kidney, and lung histology [30]. Similarly, 14-day administration at 10 mg/kg/day produced no apparent histopathological abnormalities in the heart, liver, spleen, lung, or kidney (Fig. S6A), providing preliminary evidence of *in vivo* safety at the therapeutic dose (10 mg/kg).

Costunolide, a natural anti-inflammatory sesquiterpene lactone that shares structural features with αMγB, ameliorates UC [42]. Notably, αMγB (10 mg/kg) demonstrated efficacy comparable to both costunolide and Sulfasalazine (SASP) in UC mice, with effects surpassing costunolide in certain aspects, confirming its conserved SL-derived anti-inflammatory activity and therapeutic potential (Fig. 2K–O; Fig. S3N–Z; Fig. S4A–L; Fig. S5J–M). Critically, co-administration of the Nrf2 inhibitor ML385 significantly abrogated the therapeutic effects of αMγB in the UC mouse model, indicating that the efficacy of αMγB is dependent on the Nrf2 signaling pathway (Fig. 2K–N, P; Fig. S3N-O, U-Z; Fig. S4A-B, E, G-L; Fig. S5A-B, D, L-M).

### 3.3 αMγB activates Nrf2 through p62-mediated autophagic-lysosomal degradation of Keap1

To identify potential mechanisms of αMγB in p62 regulation and Nrf2 activation, RNA sequencing was performed in αMγB-treated macrophages. The results revealed significant upregulation of Nrf2-controlled antioxidant genes (*Hmox1, Nqo1, Gclm*) (Fig. S7A). Consistently, αMγB increased both mRNA and protein levels of Nrf2 and its downstream targets (HO-1, NQO1, GCL) in a time- and dose-dependent manner, and promoted the degradation of the Nrf2 negative regulator, Keap1 (Fig. 3A–D; Fig. S7B–F). A similar upregulation of the Nrf2 pathway was observed in NCM460 colon epithelial cells and the colon tissues of UC mice (Fig. 3E; Fig. S7G–K). Moreover, αMγB not only promoted Nrf2 nuclear translocation but also enhanced its heterodimerization with the co-factor the v-MAF avian musculoaponeurotic fibrosarcoma oncogene homolog G (MAFG) (Fig. 3F–H; Fig. S7L). This complex specifically initiated transcription of downstream anti-inflammatory and antioxidant genes [43]. Conversely, the Nrf2 inhibitor ML385 completely abolished all the beneficial effects mediated by αMγB (Fig. 3I–L, V-Y; Fig. S7K, M-N). Because p62 has been reported to activate Nrf2 by binding to and sequestering Keap1 in autophagosomes [11,44], we subsequently confirmed that αMγB enhances the p62-Keap1 interaction in the presence of the autophagy inhibitor chloroquine (CQ) (Fig. 3M–N; Fig. S7O–P). Furthermore, the results confirmed that αMγB not only alleviated the LPS-induced blockade of autophagic flux but also enhanced the co-localization of p62-Keap1 with the lysosomal marker Lamp1, indicating directed degradation through autolysosomes (Fig. 3O–P). These results suggest that αMγB activates Nrf2 through p62-mediated autophagic-lysosomal degradation of Keap1, aligning with the non-canonical Nrf2 activation pathway [45].

**Fig. 3:**
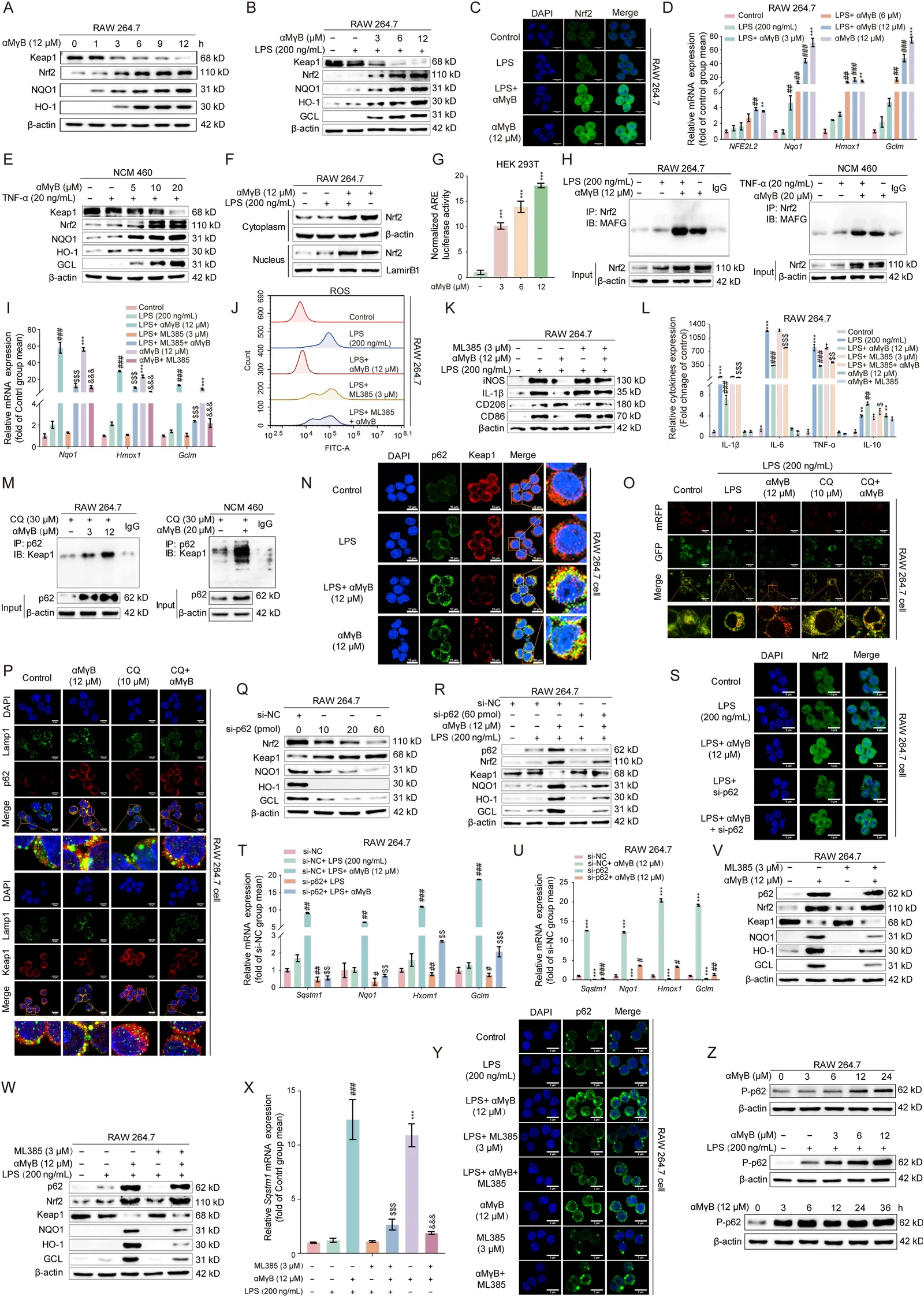
**αMγB activates Nrf2 through p62-mediated autophagic-lysosomal degradation of Keap1.** (A-E) αMγB increased both mRNA and protein levels of Nrf2 and its downstream targets (HO-1, NQO1, GCL) in a time- and dose-dependent manner and promoted the degradation of the Keap1 (Scale bar = 10 μm; n = 3). (F) αMγB enhances nuclear translocation and expression of Nrf2 (n = 3). (G) αMγB induces ARE reporter activity in HEK293T cells transfected with ARE-luciferase construct (24 h treatment; dual-luciferase assay; n = 3). (H) αMγB promotes Nrf2-MAFG interactions (n = 3). (I) ML385 reverses αMγB-mediated upregulation of Nrf2 target genes (*Nqo1*, *Hmox1*, *Gclm*) mRNA (n = 3). (J) ML385 abrogates αMγB's suppression of ROS (n = 3). (K) The ability of αMγB to suppress M1 markers (CD86, iNOS, IL-1β) and induce the M2 marker CD206 was reversed by ML385 (n = 3). (L) ML385 abrogates αMγB's suppression of pro-inflammatory cytokines (IL-1β, TNF-α, IL-6) and induction of anti-inflammatory IL-10 (n = 3). (M − N) αMγB enhances p62-Keap1 interactions and co-localization (Scale bar = 10 μm; n = 3; Chloroquine (CQ), a classic autophagy inhibitor, blocks the late stage of autophagy by inhibiting autophagosome-lysosome fusion and impairing lysosomal degradation). (O) αMγB restores LPS-impaired autophagic flux in RAW264.7 cells, reversed by CQ (Scale bar = 25 μm; n = 3). (P) αMγB enhanced co-localization of p62, Keap1 and lysosomal marker Lamp1, an effect counteracted by CQ (Scale bar = 10 μm; n = 3). (Q-U) p62 silencing abolished αMγB-mediated upregulation of p62, Nrf2, and downstream effectors (NQO1, HO-1, GCL) at protein and mRNA, and Keap1 degradation (Scale bar = 5 μm; n = 3). (V-W) ML385 abrogates αMγB-mediated upregulation of p62, Nrf2, NQO1, HO-1, GCL and Keap1 degradation (Scale bar = 5 μm; n = 3). (X-Y) ML385 antagonizes αMγB-induced *Sqstm1* mRNA and protein levels (Scale bar = 5 μm; n = 3). (Z) αMγB dose- and time-dependently enhances p62 phosphorylation (n = 3). *P < 0.05, **P < 0.01 and ***P < 0.001 vs. the Control or the si-NC, while ^*#*^*P* < 0.05, ^*##*^*P* < 0.01 and ^*###*^*P* < 0.001 vs. the LPS or the si-NC + LPS, while ^*$*^*P* < 0.05, ^*$$*^*P* < 0.01 and ^*$$$*^*P* < 0.001 vs. the LPS+ αMγB or the si-NC + LPS+ αMγB, while ^*&*^*P* < 0.05, ^*&&*^*P* < 0.01 and ^*&&&*^*P* < 0.001 vs. the αMγB.

To underscore the importance of p62 in αMγB-mediated autophagic degradation of Keap1 and Nrf2 activation, we silenced p62 in RAW264.7 cells. Si-p62 showed the highest knockdown efficiency and effectively blocked Nrf2 pathway activation (Fig. 3Q; Fig. S7Q). As expected, p62 knockdown weakened αMγB's ability to promote Keap1 degradation, activate Nrf2, and upregulate downstream targets (NQO1, HO-1, GCL) at both mRNA and protein levels (Fig. 3R–U; Fig. S7R–S; Fig. S8A–B). Consistent with prior *in vivo* studies, these results confirm that αMγB exerts its anti-inflammatory and macrophage-repolarizing effects through the p62-dependent autophagic degradation of Keap1 and subsequent activation of the Nrf2 pathway.

Since p62 is involved in the transcription of ARE elements, we investigated the influence of Nrf2 on p62 transcription and translation. Upon Nrf2 inhibition, αMγB does not affect the mRNA level of p62 but still partially maintains the expression of p62 protein (Fig. 3V–Y; Fig. S8C–E). Furthermore, p62 knockdown markedly blocked Nrf2 activation and downstream signaling (Fig. 3R–U; Fig. S7R–S; Fig. S8A–B). These results clearly demonstrate that αMγB triggers a self-reinforcing p62-Nrf2 positive feedback loop that sustains Nrf2 activation and facilitates its involvement in antioxidant and anti-inflammatory processes. Consistent with the reports that augmented phosphorylation of p62 (particularly at Ser349) enhanced its binding affinity to Keap1 [46], αMγB also facilitates Nrf2 activation by promoting the phosphorylation of p62 (Fig. 3Z; Fig. S8F–H).

### 3.4 αMγB stabilizes the expression of p62 via targeting p62 and disrupting its ubiquitination

αMγB did not affect the mRNA level of p62 but also upregulated the expression of p62 protein, suggesting the existence of an alternative, post-transcriptional regulation by αMγB. Analysis of protein degradation pathways using ubiquitin-proteasome inhibitor (MG132) and CQ, individually or collectively, revealed that αMγB does not affect the lysosomal degradation of p62 (Fig. 4A; Fig. S9A). Furthermore, cycloheximide (CHX, a protein synthesis inhibitor) pulse-chase assays demonstrated that αMγB significantly delayed p62 degradation (Fig. 4B–C). These findings indicate that αMγB enhances p62 stability primarily by suppressing its degradation through the ubiquitin-proteasome pathway. Consistent with this hypothesis, αMγB significantly reduced ubiquitination levels of p62 in both RAW264.7 and NCM460 cells (Fig. 4D; Fig. S9B).

**Fig. 4:**
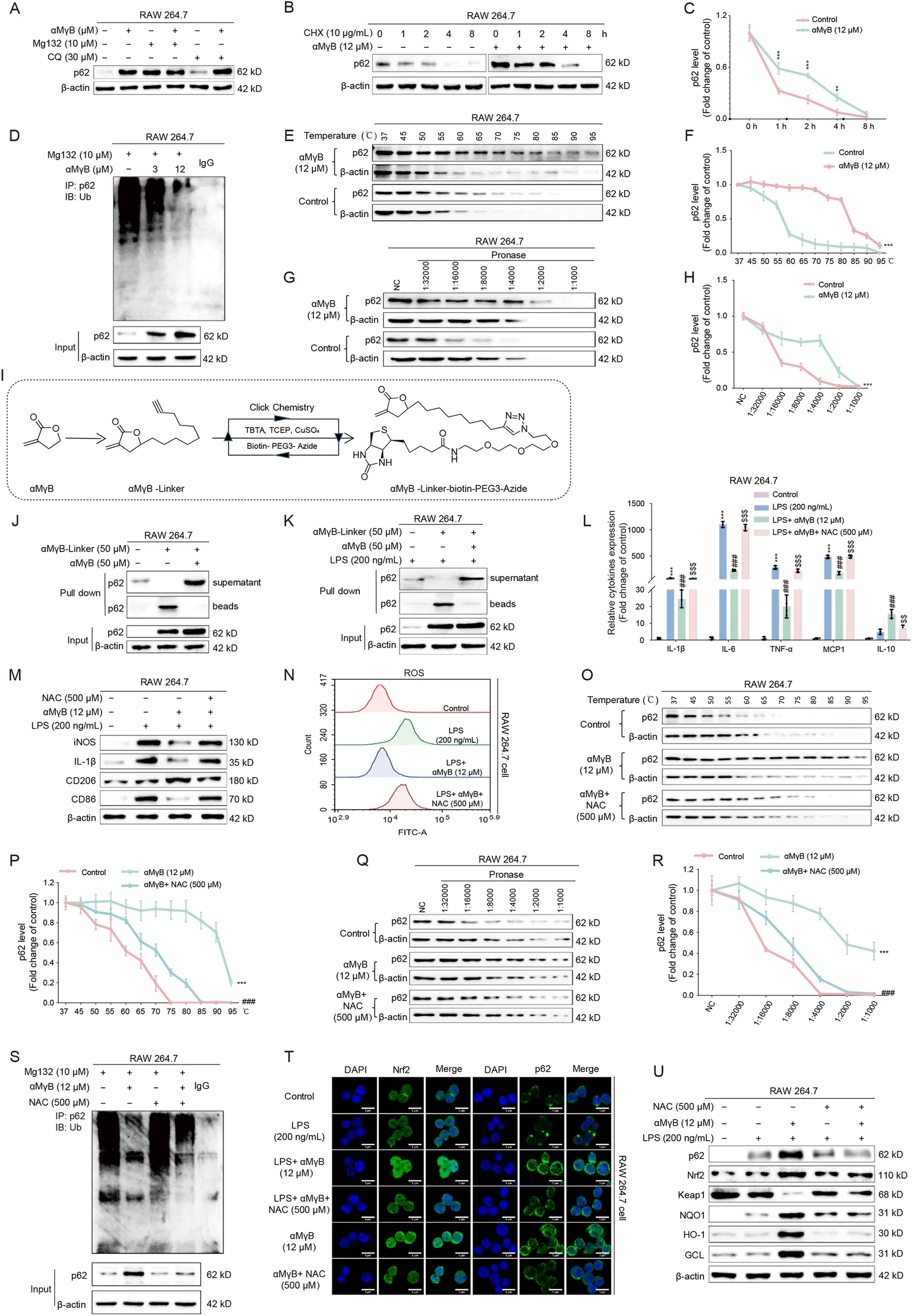
**αMγB stabilizes the expression of p62 via targeting and blocking its ubiquitination.** (A) αMγB does not affect lysosomal degradation of p62 (n = 3). (B-C) Cycloheximide chase assay demonstrates αMγB prolongs p62 half-life (n = 3). (D) αMγB enhances disruption of p62 ubiquitination (n = 3). (E-H) CETSA and DARTS show that αMγB can increase the thermal stability of p62 protein (n = 3). (I) αMγB-Linker design and click chemistry conjugation strategy. (J-K) Pull-down assays show αMγB-Linker captures p62 with reduced affinity versus native αMγB (n = 3). (L-N) NAC abrogates αMγB's suppression of ROS, pro-inflammatory cytokines and M1 markers (CD86, iNOS, IL-1β, TNF-α, IL-6, MCP-1) and induction of M2 markers (CD206, IL-10) (n = 3). (O-R) NAC disrupts direct αMγB-p62 binding (validated by CETSA and DARTS) (n = 3). (S) NAC abolishes αMγB-mediated disruption of p62 ubiquitination (n = 3). (T-U) NAC reversed the αMγB-induced upregulation of p62, Nrf2, and downstream proteins, as well as Keap1 degradation (Scale bar = 5 μm; n = 3). *P < 0.05, **P < 0.01 and ***P < 0.001 vs. the Control, while ^*#*^*P* < 0.05, ^*##*^*P* < 0.01 and ^*###*^*P* < 0.001 vs. the LPS, while ^*$*^*P* < 0.05, ^*$$*^*P* < 0.01 and ^*$$$*^*P* < 0.001 vs. the LPS+ αMγB.

To determine whether deubiquitination enzymes (DUBs) contributed to the αMγB-induced reduction in p62 ubiquitination, we examined the involvement of specific DUBs. To this end, we screened the UbiBrowser database (http://ubibrowser.bio-it.cn/) and identified several high-confidence candidates, including ZRANB1, USP15, and USP20 in humans, as well as Usp8 and Zranb1 in mice (Fig. S9C). However, qPCR analysis showed that αMγB did not alter the mRNA levels of these DUBs (Fig. S9D–E). Furthermore, focusing on the highly conserved ZRANB1 (Zranb1) across human and murine species, Western blot and Co-IP assays revealed that αMγB neither affected its protein expression nor its interaction with p62 (Fig. S9F–H). Collectively, these data suggest that the regulatory effect of αMγB on p62 ubiquitination is unlikely to be mediated through these specific DUBs.

Secondary mass spectrometry analysis in quantitative proteomics revealed αMγB covalently bound to four cysteine residues (C219, C113, C26, and C291) on p62, suggesting that p62 is a direct target (Table S4). This result was further validated by CETSA and DARTS assays (Fig. 4E–H). To definitively establish p62 as a direct target of αMγB, we synthesized an αMγB-Linker probe (Fig. 4I). Then, affinity pulldown assays employing click chemistry with this probe successfully captured p62 from cell lysates, confirming a direct binding interaction (Fig. 4J–K). Additionally, co-treatment with N-acetylcysteine (NAC), which possesses a more reactive sulfhydryl group than p62's cysteine residues, abolished αMγB's ability to modulate macrophage polarization. Specifically, αMγB could no longer suppress LPS-induced ROS, NO, pro-inflammatory cytokines, and M1 markers (iNOS, IL-1β, CD86, IL-6, TNF-α, MCP-1) or promote M2 markers (CD206, IL-10) in RAW264.7 macrophages (Fig. 4L–N; Fig. S9I–K). Moreover, NAC disrupted the αMγB-p62 binding and abolished αMγB-induced disruption of p62 ubiquitination, thereby blocking the subsequent autolysosomal degradation of Keap1 and activation of the Nrf2 signaling pathway (Fig. 4O–U; Fig. S9L–O). These results indicate that through direct interaction with the sulfhydryl groups of p62's cysteine residues, αMγB promotes disruption of p62 ubiquitination and stabilization, which results in the activation of the Nrf2 signaling pathway and the sustained anti-inflammatory and antioxidant effects.

### 3.5 αMγB prevents the exposure of ubiquitination sites at position 297 by alkylating cysteine 291

To elucidate how αMγB reduces p62 ubiquitination and thereby stabilizes p62 to exert anti-inflammatory effects, we performed molecular-docking simulations to examine its covalent interactions with the four candidate cysteine residues (Fig. S10A–L). Notably, our consultation of the PhosphoSitePlus® database indicated that Lys297 plays a critical role in E3 ubiquitin ligase recruitment and p62 ubiquitination (Fig. 5A). Molecular docking revealed that binding of αMγB to C291, given its proximity to Lys297, may spatially hinder the ubiquitination site at Lys297 and disrupt the p62-E3 interface, thereby impeding E3 ligase recruitment (Fig. 5B). The E3 ubiquitin ligase TRIM21, which binds and ubiquitinates p62 to disrupt its sequestration function, was further validated as a target of this mechanism, with αMγB treatment reducing p62-TRIM21 complex formation [47] (Fig. 5C–D).

**Fig. 5:**
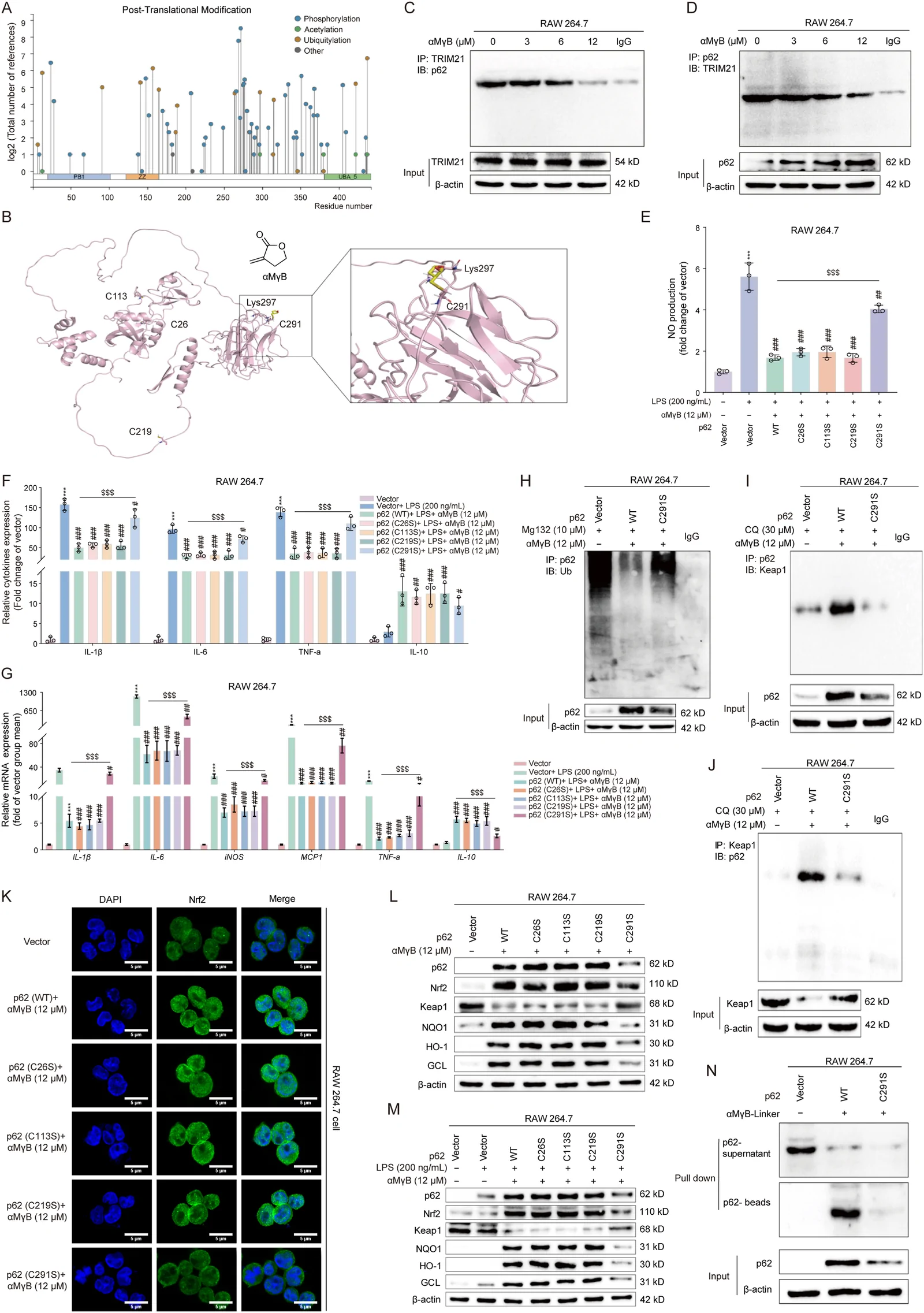
**αMγB prevents exposure of 297-E3-linked ubiquitin sites by alkylation of C291.** (A) The PhosphoSitePlus® database demonstrates that the Lys297 site is critical for p62 ubiquitination. (B) Molecular docking indicates that αMγB sterically hinders the interaction between p62 and the E3 ubiquitin ligase TRIM21. (C-D) αMγB prevents the binding of p62 to TRIM21 (n = 3). (E-G) In LPS-induced RAW264.7 macrophage inflammation models, αMγB fails to inhibit NO and pro-inflammatory cytokine release, M1 polarization markers (TNF-α, IL-1β, IL-6, MCP-1, iNOS), or promote anti-inflammatory cytokine and M2 marker (IL-10) expression following the p62 (C291S) mutation (n = 3). (H) αMγB loses its ability to induce disruption of p62 ubiquitination upon the p62 (C291S) mutation (n = 3). (I-J) The αMγB-mediated promotion of p62-Keap1 interaction is partially abolished by the p62 (C291S) mutation (n = 3). (K-M) p62 (C291S) mutation partially blocks αMγB-induced activation of the p62-Nrf2 signaling pathway (Scale bar = 5 μm; n = 3). (N) αMγB-Linker (50 μM) fails to pull down p62 protein after p62 (C291S) mutation (n = 3). *P < 0.05, **P < 0.01 and ***P < 0.001 vs. the Vector, while ^*#*^*P* < 0.05, ^*##*^*P* < 0.01 and ^*###*^*P* < 0.001 vs. the Vector + LPS, while ^*$*^*P* < 0.05, ^*$$*^*P* < 0.01 and ^*$$$*^*P* < 0.001 vs. the p62 (WT) + LPS + αMγB.

We generated p62-WT and four mutants from cysteine to serine (C26S, C113S, C219S, C291S), confirming comparable transfection efficiency and stable expression for all constructs (Fig. S10M–N). In contrast to mutations at other residues, mutating the p62 (C291S) site markedly impaired the effect of αMγB on suppressing NO production, pro-inflammatory cytokine release, M1 polarization markers, and enhancing IL-10 expression in LPS-stimulated RAW264.7 macrophages (Fig. 5E–G). Additionally, mutation at the p62 (C291S) site partially abolished αMγB-mediated disruption of p62 ubiquitination. The C291S mutation also impaired αMγB-enhanced p62-Keap1 binding, thereby preventing p62 stabilization, autolysosomal degradation of Keap1, and subsequent activation of the Nrf2 signaling pathway (Fig. 5H–M; Fig. S10O–Q). Affinity-based target pull-down assays further confirmed that this mutation partially prevented αMγB binding to p62 (Fig. 5N). Taken together, our data show that αMγB alkylates p62 at C291 to block its ubiquitination and promote stabilization, thereby inducing Keap1 degradation and Nrf2 release, ultimately executing its anti-inflammatory function.

### 3.6 αMγB fails to inhibit macrophage M1 polarization and alleviate UC in *Sqstm1*^C291S^ mutant mice

To further elucidate the crucial role of p62 (C291) in αMγB-mediated anti-inflammatory therapy *in vivo*, we administered AAV9-*Sqstm1* and AAV9-*Sqstm1* (C291S) by intracolonic delivery to p62-knockout mice and established a DSS-induced UC model (Fig. 6A). In *Sqstm1*^−/−^ knockout mice, re-expression of *Sqstm1*^WT^ restored αMγB's ability to ameliorate DSS-induced pathologies, as evidenced by the reversal of shortening and body-weight loss, reduced spleen index and DAI, and restored colonic integrity. In contrast, *Sqstm1*^C291S^ mutant overexpression failed to restore these protective effects (Fig. 6B–E; Fig. S11A–B). ELISA corroborated these findings (Fig. 6F; Fig. S11C). In addition, αMγB lost its ability to protect the intestinal barrier through tight junction protein upregulation (Occludin 1, Claudin 1, ZO-1) in *Sqstm1*^−/−^ knockout UC mice. Reconstitution with *Sqstm1*^WT^ restored barrier protection, whereas *Sqstm1*^C291S^ mutant overexpression failed to rescue this function (Fig. 6G; Fig. S11D). These findings establish the C291 residue as the critical target through which αMγB regulates p62-mediated intestinal barrier protection.

**Fig. 6:**
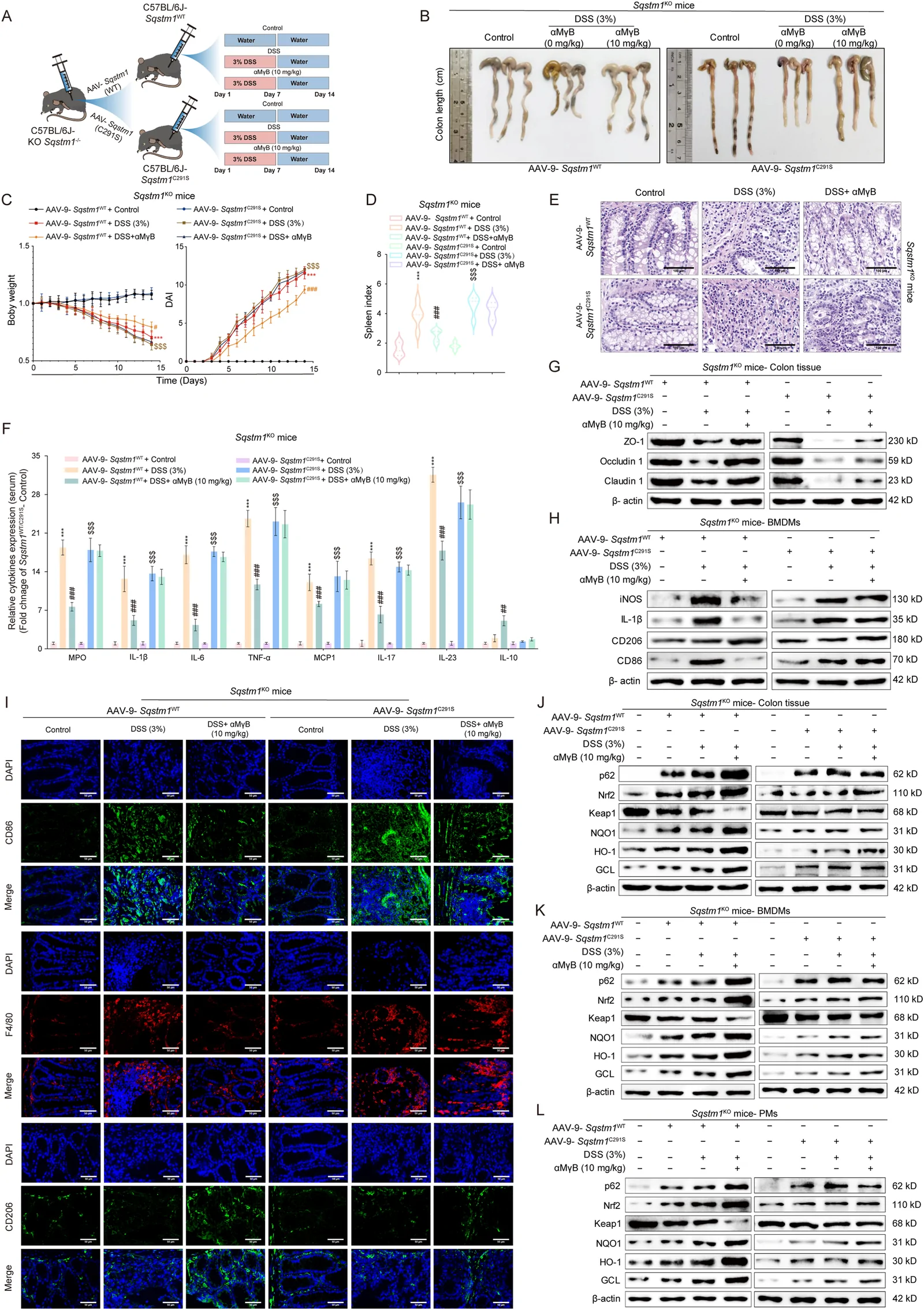
**αMγB fails to inhibit macrophage M1 polarization and alleviate UC in *Sqstm1*^C291S^ mutant mice.** (A) Schematic of establishing UC models in *Sqstm1*^−/−^ knockout mice reconstituted with either *Sqstm1*^WT^ or *Sqstm1*^C291S^ mutant overexpression. (B) Colon length across experimental groups (n = 5). (C) Body weight changes and DAI scores (n = 5). (D) Spleen index measurements (n = 5). (E) Histological analysis of colon tissue sections (H&E staining) (Scale bar = 100 μm; n = 5). (F) In DSS-induced UC using *Sqstm1*^−/−^ knockout mice, *Sqstm1*^WT^ reconstitution restored αMγB's ability to suppress serum pro-inflammatory mediators (IL-6, IL-1β, TNF-α, MCP-1, IL-17, IL-23, MPO) and enhance IL-10 expression. This rescue was partially abolished by *Sqstm1*^C291S^ overexpression (n = 5). (G) Reconstitution of *Sqstm1*^WT^ restored the ability of αMγB to maintain the expression of colonic tight junction proteins (Occludin 1, Claudin 1, ZO-1) in UC mice, whereas overexpression of the *Sqstm1*^C291S^ mutant failed to rescue this function (n = 5). (H-I) Reconstitution of *Sqstm1*^WT^ fully restored the regulatory effects of αMγB on the polarization imbalance in macrophages (BMDMs and PMs), whereas the *Sqstm1*^C291S^ mutant failed to rescue these effects (Scale bar = 50 μm; n = 5). (J-L) Reconstitution of *Sqstm1*^WT^ fully restored the activation of the p62-Keap1-Nrf2 pathway by αMγB, whereas overexpression of the *Sqstm1*^C291S^ mutant failed to rescue this activation (n = 5). *P < 0.05, **P < 0.01 and ***P < 0.001 vs. the AAV-9- *Sqstm1*^WT^ - Control, while ^*#*^*P* < 0.05, ^*##*^*P* < 0.01 and ^*###*^*P* < 0.001 vs. the AAV-9- *Sqstm1*^WT^ - DSS (3%), while ^*$*^*P* < 0.05, ^*$$*^*P* < 0.01 and ^*$$$*^*P* < 0.001 vs. the AAV-9- *Sqstm1*^C291S^ - Control.

Genetic reconstitution with wild-type p62, but not the C291S mutant, restored the effect of αMγB on macrophage polarization, demonstrating that p62 C291 was essential for this activity. In *Sqstm1*^−/−^ knockout UC mice, αMγB failed to correct M1/M2 imbalance in BMDMs/PMs and macrophage infiltration into the colon. While *Sqstm1*^WT^ reconstitution fully restored αMγB's regulatory function, the *Sqstm1*^C291S^ mutant lost this capacity (Fig. 6H–I; Fig. S11F–J). Moreover, in *Sqstm1*^−/−^ knockout UC mice, αMγB failed to trigger Keap1 degradation and subsequent Nrf2 activation. While reintroducing *Sqstm1*^WT^ restored αMγB's efficacy, overexpression of a *Sqstm1*^C291S^ mutant partially abolished it (Fig. 6J–L; Fig. S12A–C). Together, these findings demonstrate that by targeting p62 at C291, αMγB stabilizes p62 via disruption of p62 ubiquitination, leading to Keap1 degradation, Nrf2 activation, and ultimately suppression of M1 polarization and UC pathology.

## 4 Discussion

Ulcerative colitis (UC) is characterized by chronic mucosal damage, dysregulated macrophage activation, epithelial barrier dysfunction, and persistent oxidative stress [[48], [49], [50]]. In this study, we show that targeted enhancement of p62 stability via covalent modification at the C291 residue offers an effective therapeutic strategy to break the self-reinforcing cycle of inflammation and barrier disruption in UC. Rather than acting as a non-specific antioxidant, αMγB specifically disrupts p62 ubiquitination, which in turn triggers a self-amplifying p62-Keap1-Nrf2 feedback loop. These findings directly address the critical gap in UC therapy by demonstrating that stabilizing p62 function, rather than preventing pathological p62 accumulation, can reprogram macrophage polarization from M1 to M2 and restore colonic mucosal homeostasis [39,41].

Our results accord with previous studies showing that activation of the Nrf2/Keap1 pathway confers cytoprotection against colonic inflammation and that natural products can activate p62-Nrf2 signaling or promote Keap1 degradation in other inflammatory models [51,52]. However, unlike conventional mechanisms of Nrf2 activation, the mechanism identified here is driven by p62 stabilization at C291. Mechanistically, αMγB covalently modifies C291 of p62 and may thereby sterically hinder ubiquitination at the adjacent Lys297 residue, stabilizing p62 and promoting lysosomal degradation of Keap1. The loss of therapeutic efficacy in *Sqstm1*^C291S^ mutant mice and Nrf2-inhibited models confirms that C291 modification is indispensable for αMγB's protective activity. Furthermore, while previous anti-inflammatory interventions, such as costunolide, broadly target NF-κB signaling via electrophilic binding to IKKβ [53], αMγB exhibits superior target specificity and enhanced capacity for mucosal barrier repair.

In UC, macrophage-derived cytokines and ROS/NO disrupt epithelial tight junctions and induce epithelial MCP-1 production, thereby promoting monocyte recruitment and sustaining inflammation [40,41]. The co-culture experiments showed that conditioned medium from αMγB-treated macrophages elicited weaker cytokine and chemokine responses in epithelial cells, whereas conditioned medium from αMγB-treated epithelial cells suppressed macrophage activation and increased macrophage IL-10 expression. Thus, αMγB attenuates both macrophage-to-epithelial injury and epithelial-to-macrophage feedback, thereby breaking the self-reinforcing inflammatory loop and highlighting its coordinated dual-cell activity. Longstanding UC promotes colitis-associated CRC through recurrent epithelial injury, inflammatory and oxidative DNA damage, barrier dysfunction, and microbial translocation [54,55]. By stabilizing p62, promoting Keap1 degradation, and activating Nrf2 signaling, αMγB suppresses M1 macrophage polarization and pro-inflammatory cytokine and ROS production, restores epithelial tight-junction integrity, and may thereby reduce the pro-tumorigenic inflammatory and genotoxic microenvironment that drives colitis-associated carcinogenesis [56,57]. The effects of αMγB on the gut microbiota likely arise largely from its anti-inflammatory and barrier-restorative actions. αMγB suppresses M1-like macrophage activation and the associated production of pro-inflammatory cytokines and ROS, thereby attenuating epithelial inflammation and restoring tight junction integrity. The resulting improvement in barrier function may limit bacterial translocation and favor the recovery of beneficial bacteria. In turn, microbiota-derived metabolites may reinforce epithelial repair and immune homeostasis. Thus, microbiota remodeling may initially arise as a consequence of mucosal healing and subsequently contribute to maintaining the therapeutic response to αMγB [56]. In line with this interpretation, reduced inflammation and improved barrier function have been associated with changes in the gut microbiota and its metabolites [58,59].

Nonetheless, the present findings must be interpreted in light of several limitations. First, although structural modeling suggests that αMγB binding at C291 may sterically restrict ubiquitination of the adjacent Lys297 residue, this proposed mechanism requires direct validation by site-directed mutagenesis. Second, while the *Sqstm1*^C291S^ mouse model confirms p62 as the primary target in colitis, the potential reactivity of the α,β-unsaturated ketone moiety toward other cysteine residues across the proteome requires further proteome-wide selectivity profiling [60]. Accordingly, LC-MS/MS-based cysteine-targeting screens may facilitate such proteome-wide evaluation [61,62]. Third, the acute DSS model is informative for evaluating epithelial injury and innate inflammatory responses but does not fully recapitulate the chronic, relapsing, and adaptive immune features of human UC. Future studies should therefore evaluate αMγB in chronic or relapsing models and in complementary immune-driven models, such as IL-10-deficient mice and T-cell transfer colitis.

In summary, this study identifies covalent disruption of p62 ubiquitination as a druggable mechanism for UC treatment. By establishing a self-sustaining p62-Nrf2 antioxidant loop, αMγB reprograms macrophage polarization and restores colonic barrier integrity. Future studies should determine whether colon-targeted delivery systems can further optimize the pharmacokinetic profile and therapeutic index of αMγB in clinical settings. These findings support further evaluation of αMγB as a candidate compound for UC treatment and investigation of the potential effects of αMγB on the progression of colitis-associated CRC.

## CRediT authorship contribution statement

**Ke-Gang Linghu:** Writing – review & editing, Visualization, Supervision, Methodology, Investigation. **Dasong Wang:** Writing – review & editing, Writing – original draft, Methodology, Investigation. **Tian Zhang:** Writing – review & editing, Visualization, Validation. **Fei Jiang:** Methodology. **Long Wu:** Methodology. **Di Pan:** Methodology. **Yan Chen:** Software. **Ling Tao:** Methodology. **Ligen Lin:** Resources, Methodology. **Hua Yu:** Methodology. **Qianming Du:** Visualization, Validation, Methodology. **Weidong Pan:** Visualization, Validation, Supervision, Software. **Xiangchun Shen:** Visualization, Validation, Supervision, Resources.

## Declaration of competing interest

The authors declare that they have no conflict of interest.

## Figures and Tables

**figs1:**
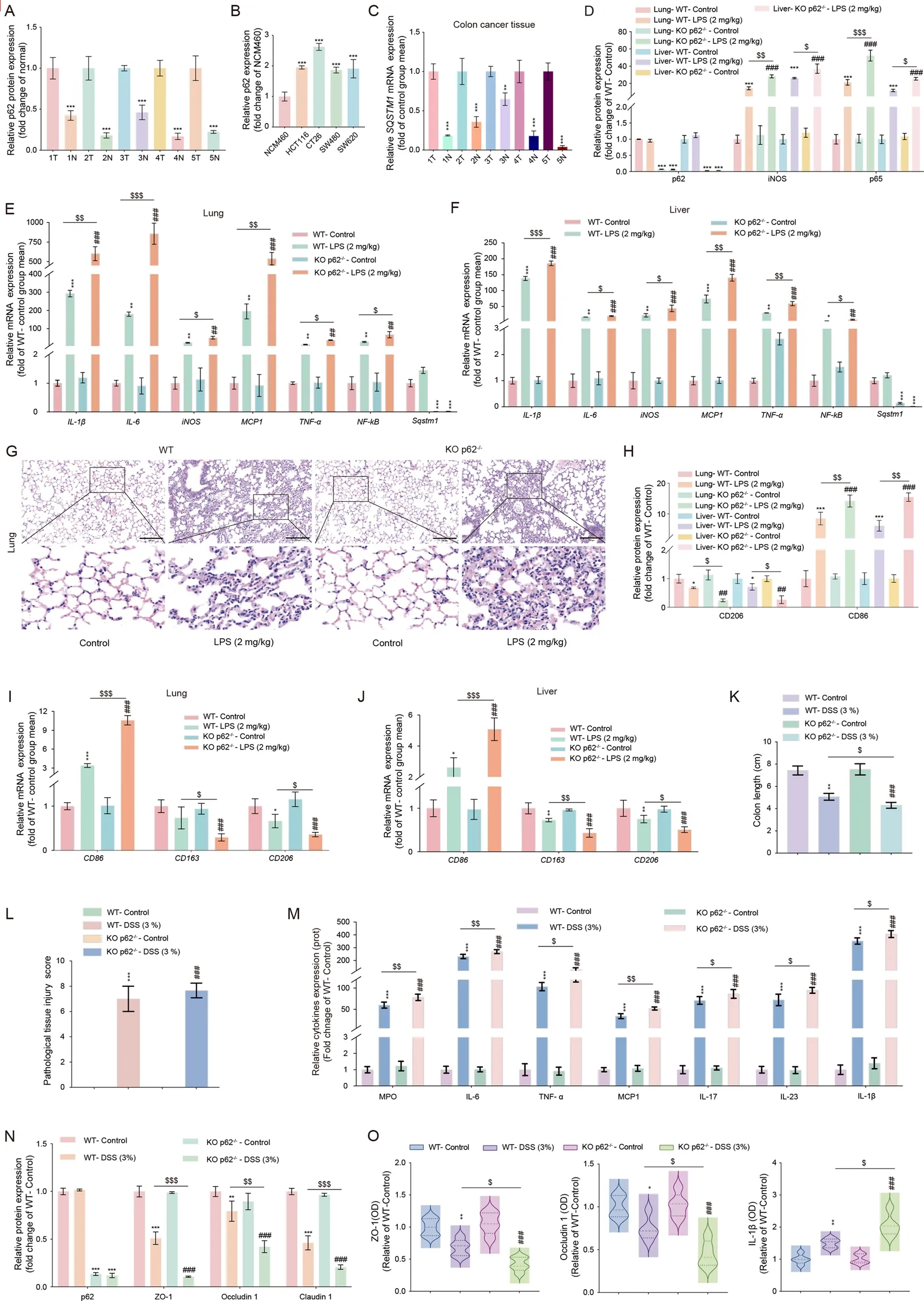


**figs2:**
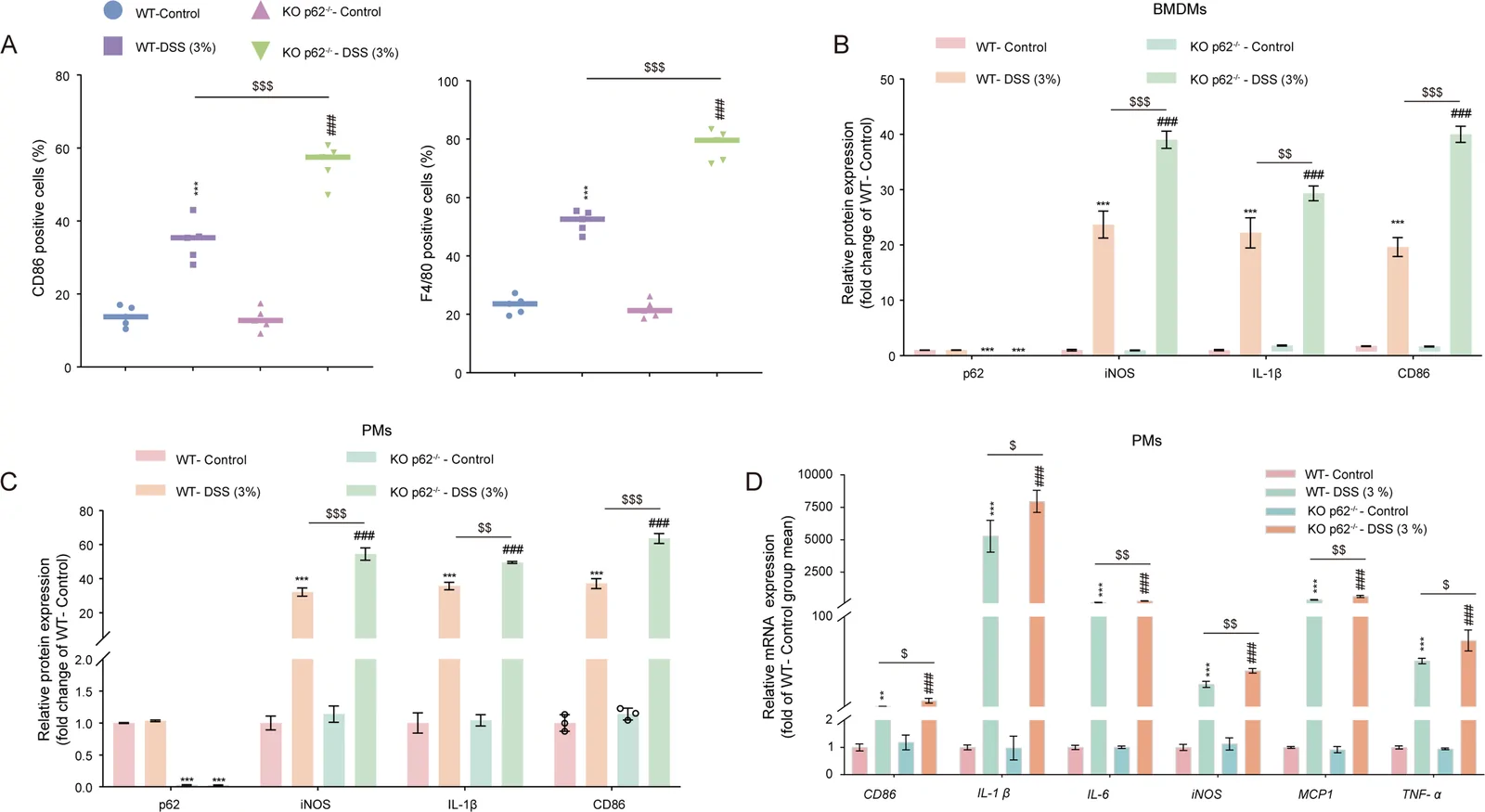


**figs3:**
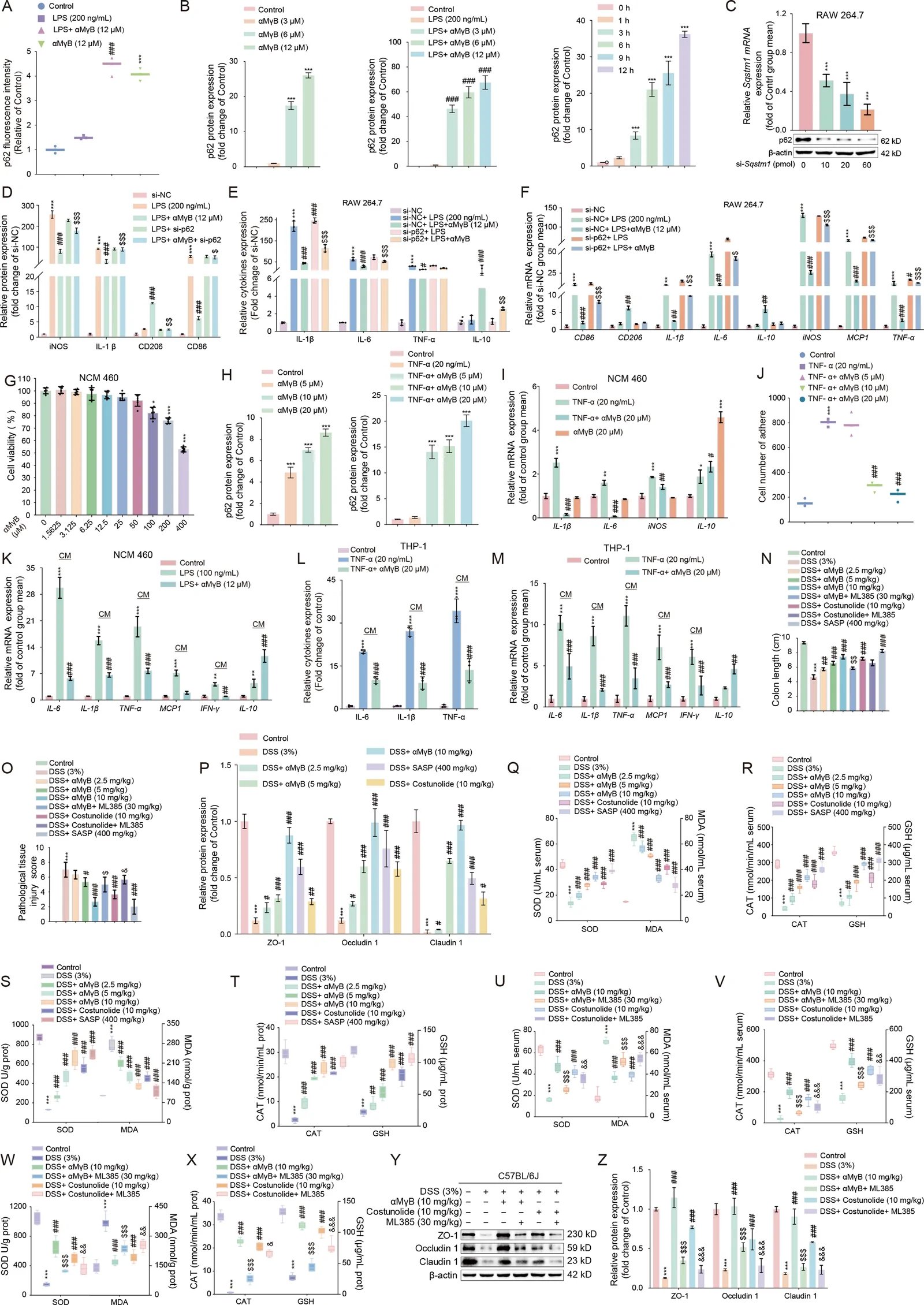


**figs4:**
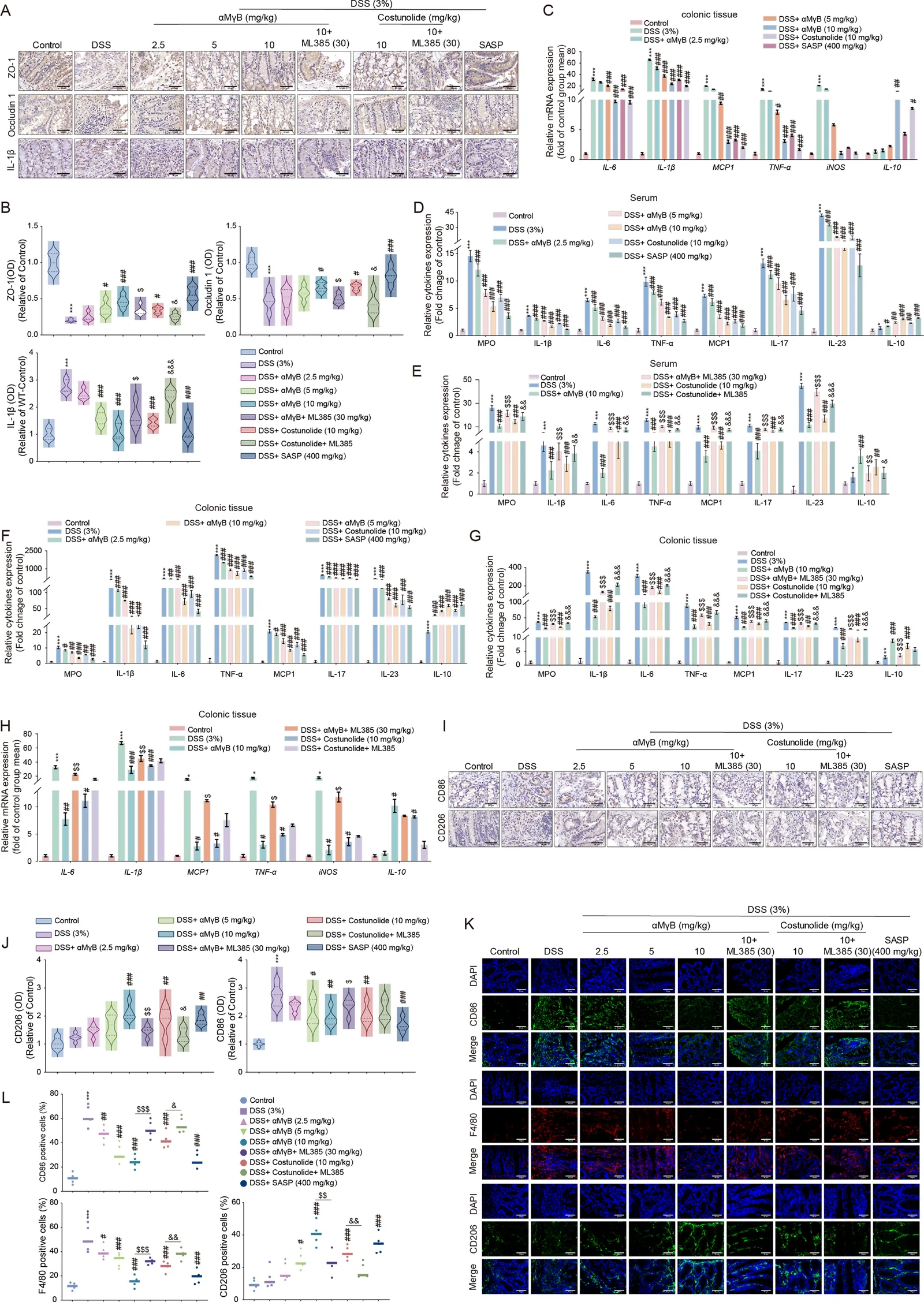


**figs5:**
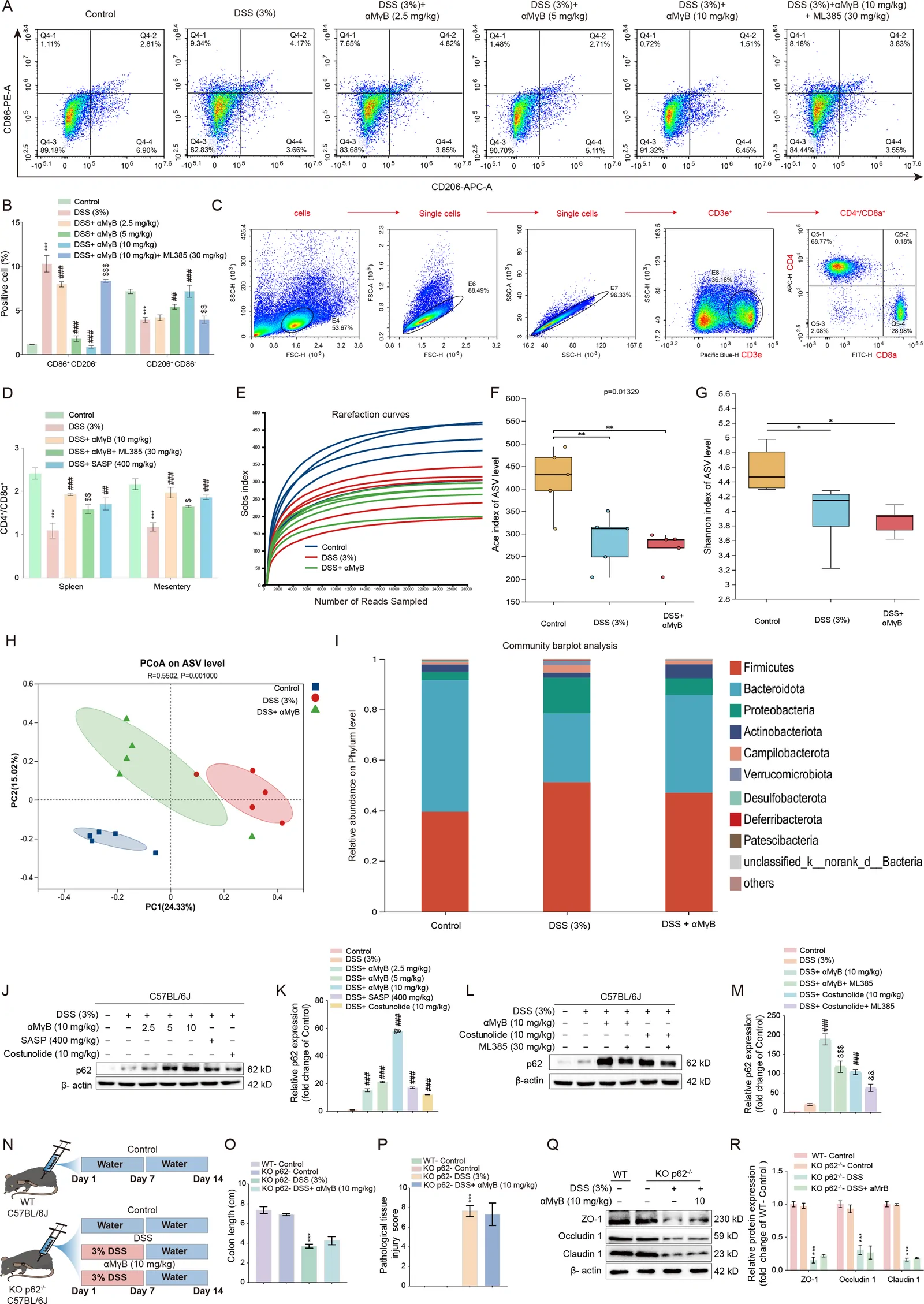


**figs6:**
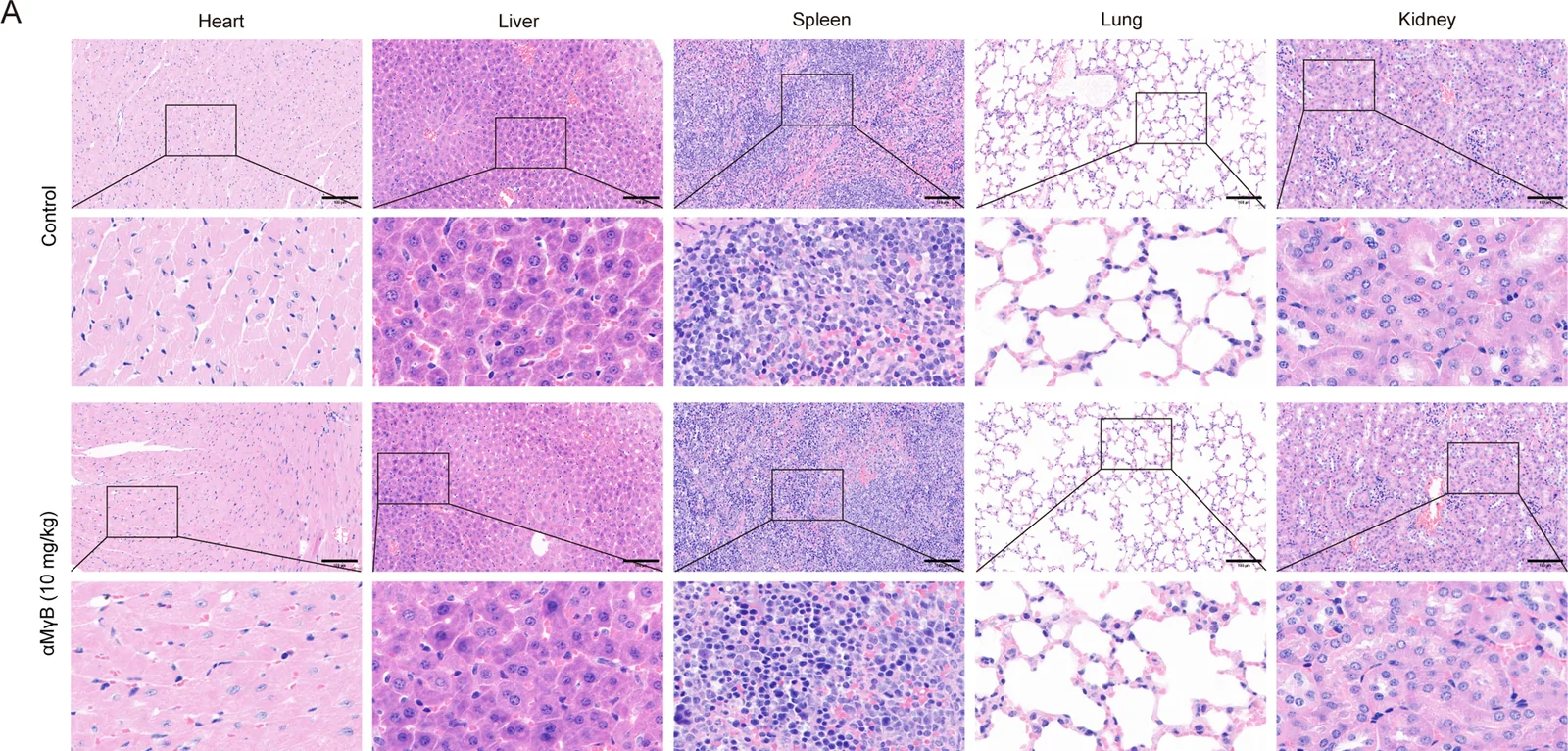


**figs7:**
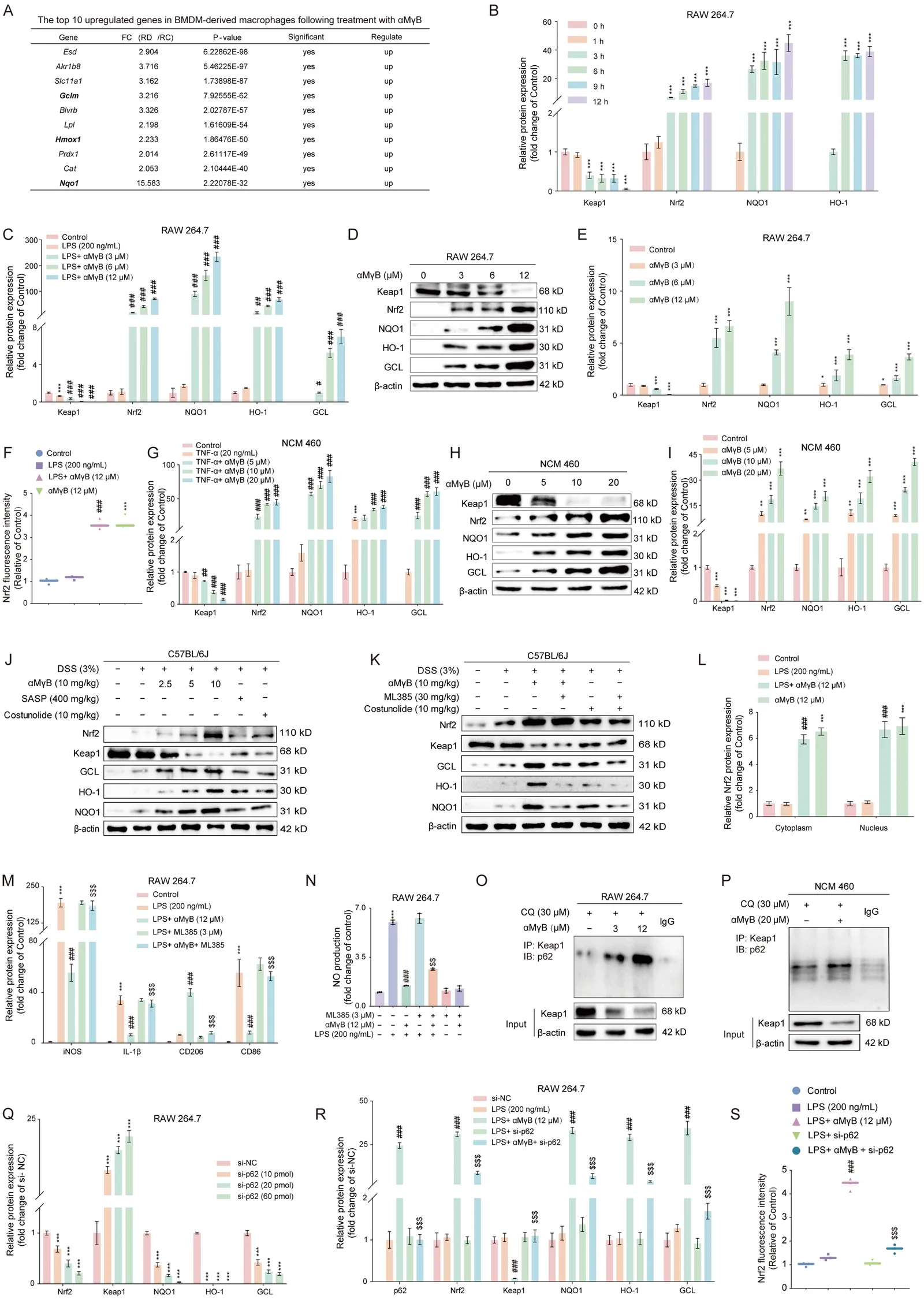


**figs8:**
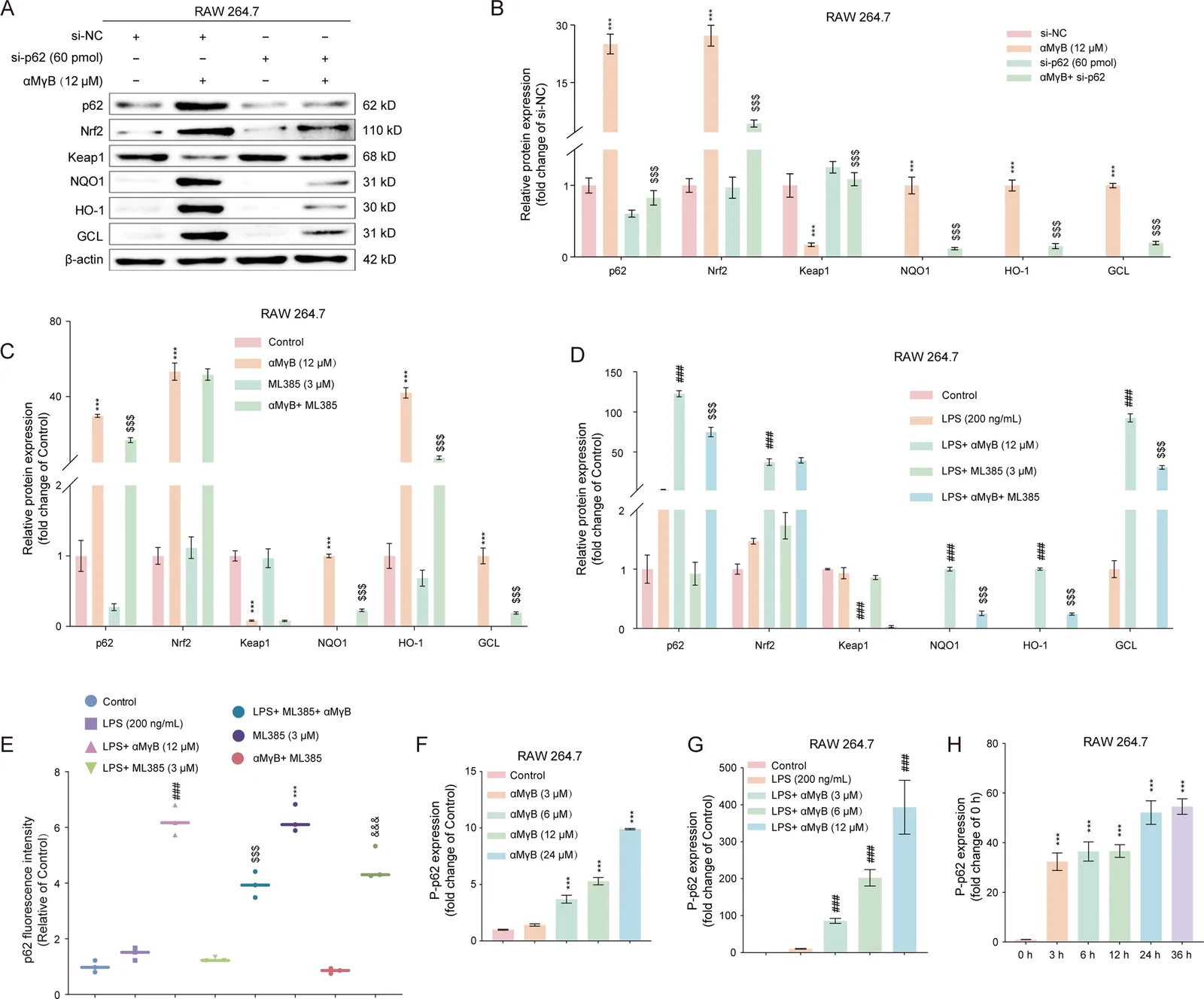


**figs9:**
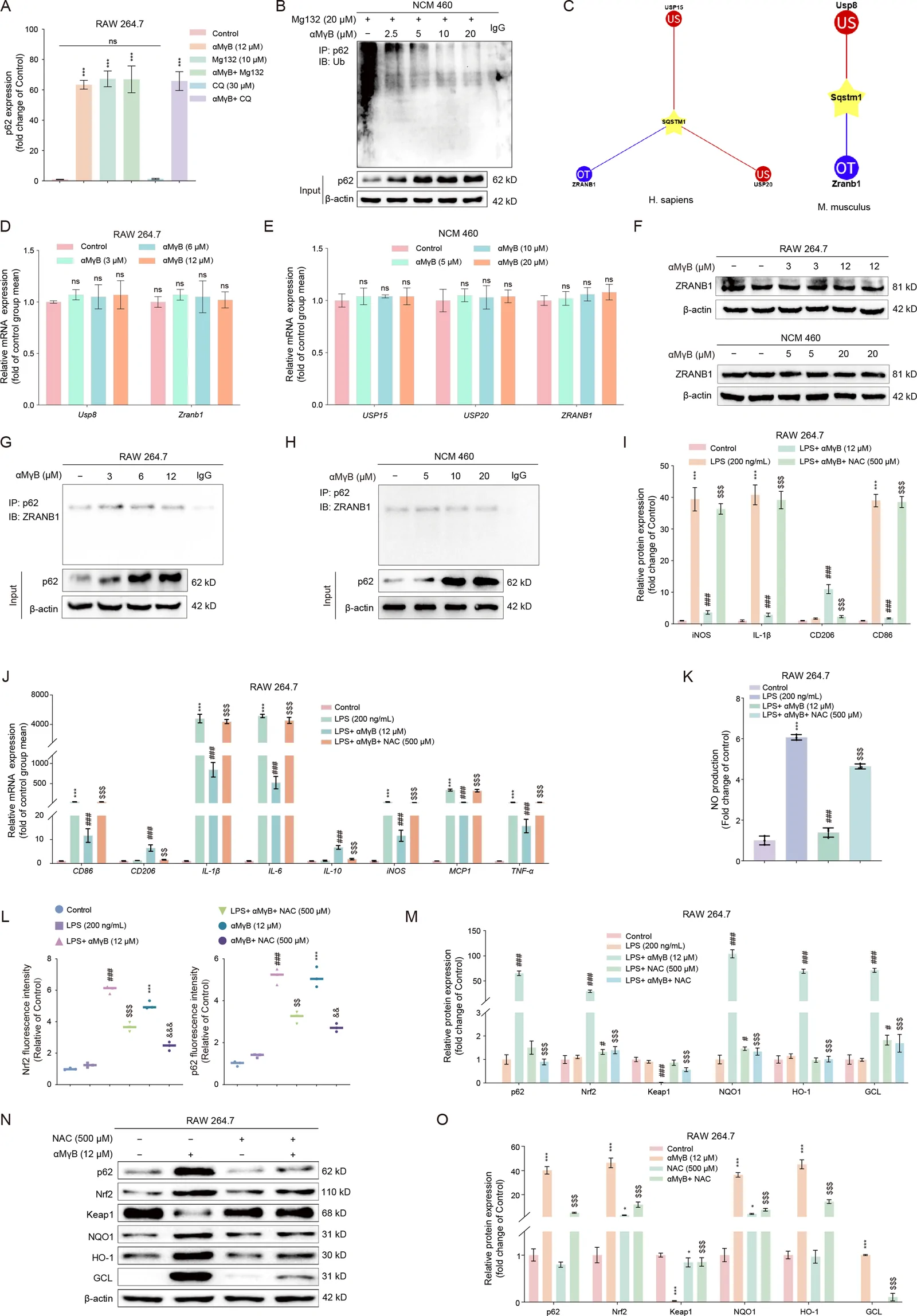


**figs10:**
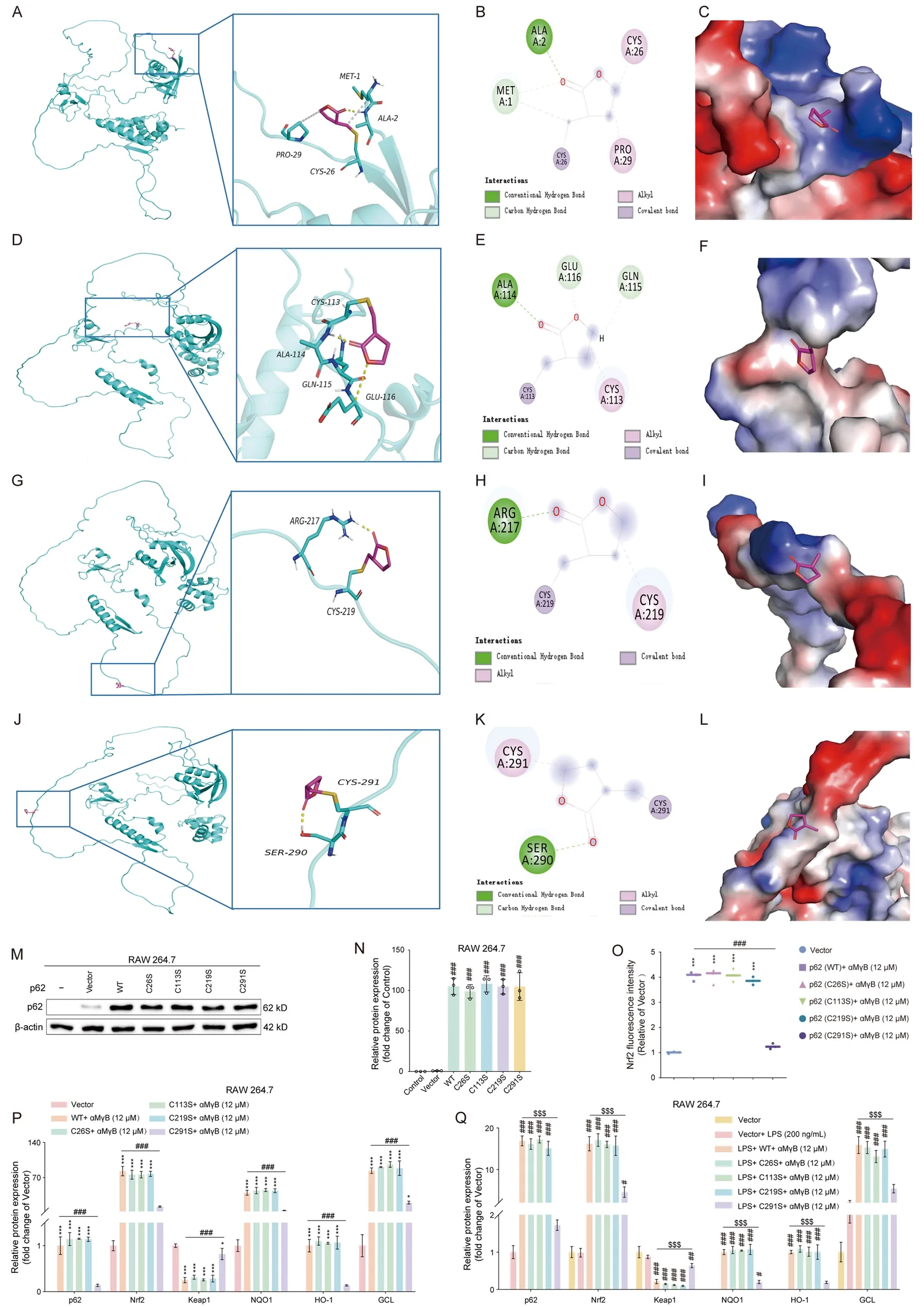


**figs11:**
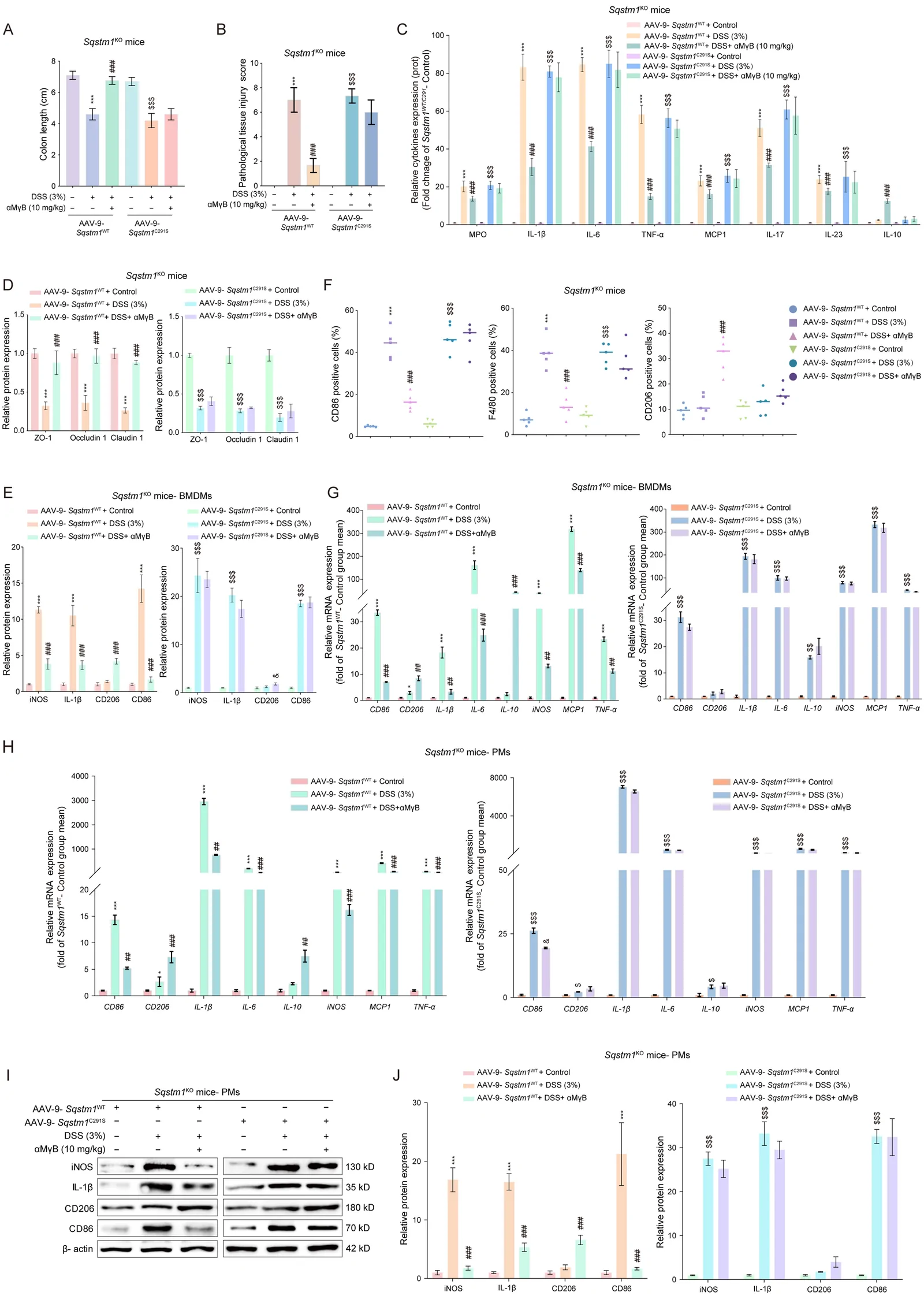


**figs12:**
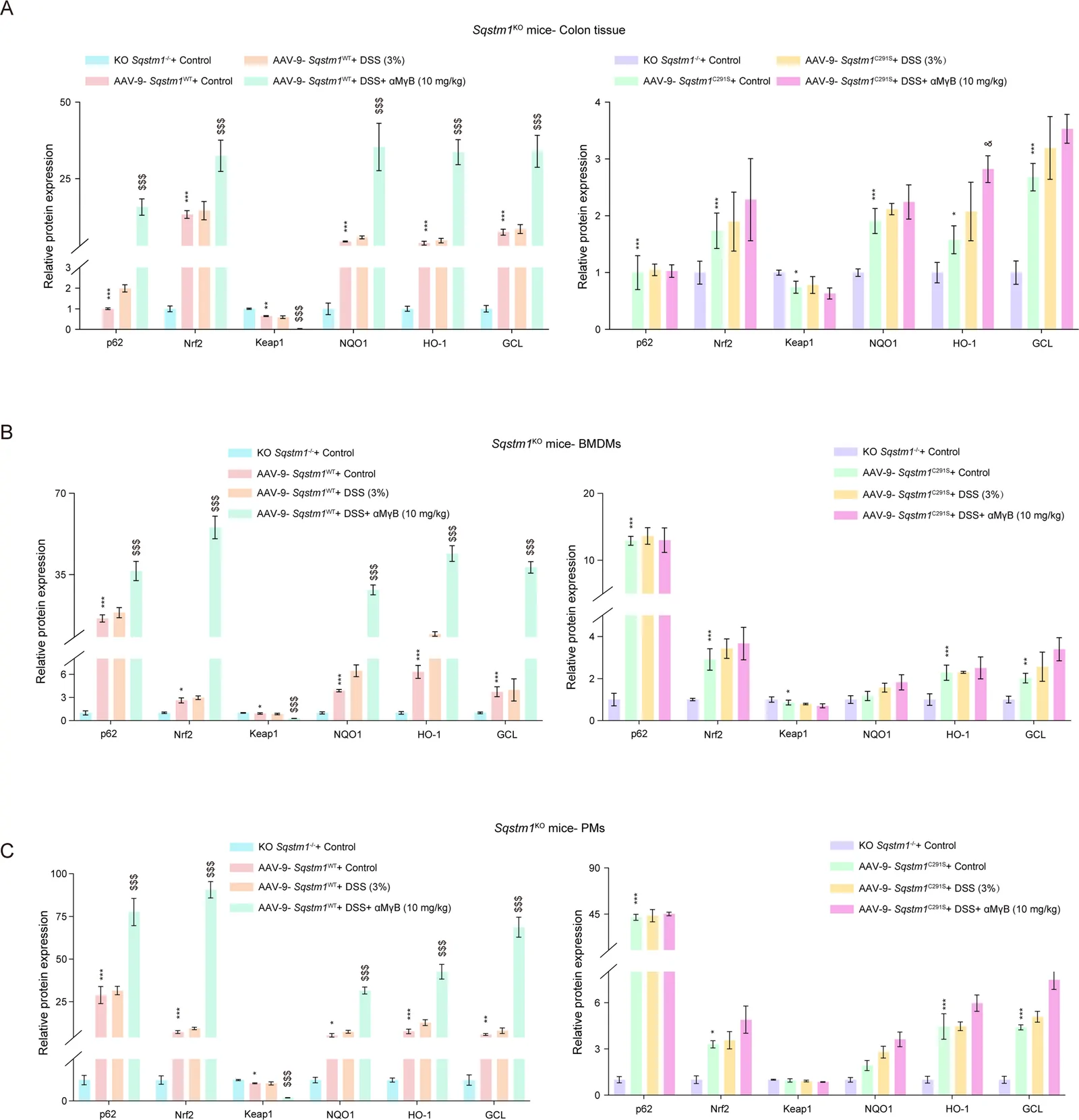


## Data Availability

Data will be made available on request.
