# Supplementary material for: Disruption of p62 ubiquitination by α-methylene-γ-butyrolactone enhances Keap1 lysosomal degradation for ulcerative colitis therapy
Source: Redox Biol. 2026 Oct 3;97:104424. doi: 10.1016/j.redox.2026.104424 (PMC13675664; doi:10.1016/j.redox.2026.104424)
Supplement: Supplementary file 1 [file mmc1.docx]

**Supporting Information**

**Table S1.** Chemical and biological materials

| **Name** | **Source** | **Identifier** |
| --- | --- | --- |
| α-Methylene-γ-butyrolactone (α-M-γ-B) | Sigma | #226416 |
| 3-(4,5-dimethylthiazol-2-yl)-2,5-diphenyl 2H-tetrazolium bromide (MTT) | Solarbio | #IM02808 |
| Lipopolysaccharides (LPS) | Sigma | #L4391 |
| N-acetylcysteine (NAC) | Sigma | #1009005 |
| Phorbol 12-myristate 13-acetate (PMA) | Sigma | #P8139 |
| DMSO | Sigma | #D1435 |
| Griess reagent | Sigma | #G4410 |
| Phosphate-buffered saline (PBS) | Solarbio | #P1010 |
| BCA Protein Assay Kit | Solarbio | #PC0020 |
| Fetal bovine serum (FBS) | Thermo | #3A5669501 |
| 2',7'-bis-(2-carboxyethyl)-5-(and-6) Carboxyfluorescein acetoxymethyl ester (BCECF/AM) | Beyotime | #S1006 |
| DMEM medium | Thermo | #21010046 |
| RPMI 1640 Medium | Thermo | # 11875119 |
| RIPA buffer (high) | Solarbio | #R0010 |
| TNF-α ELISA kit (Mouse) | Neobioscience (Neo) | #EMC102a.96 |
| TRIzol reagent | Thermo | #15596026 |
| Penicillin-streptomycin solution (P/S) | Thermo | #15140122 |
| Phenylmethylsulfonyl fluoride (PMSF) | Thermo | #36978 |
| Mounting Medium，antifading (with DAPI) | Solarbio | #S2110 |
| H&E solution | Solarbio | #G1005 |
| SYBR Green qPCR Mix | MCE | #HY-K0501 |
| RT Master Mix for qPCR II | MCE | #HY-K0510A |
| Red Blood Cell Lysis Buffer | Solarbio | #R1010 |
| MG-132 | MCE | #HY-13259 |
| Cycloheximide (CHX) | MCE | # HY-12320 |
| ML385 | MCE | #HY-100523 |
| Chloroquine (CQ) | MCE | #HY-17589A |
| Sulfasalazine (SASP) | MCE | #HY-14655 |
| Protease Inhibitor Cocktail | MCE | # HY-K0010 |
| Tris (2-carboxyethyl) phosphine hydrochloride (TCEP) | Macklin | #C4706 |
| N-acetylcysteine (NAC) | Macklin | #A0737 |
| CuSO4 | Macklin | #C805233 |
| CaCl2 | Macklin | #C805228 |
| biotin-PEG3-azide | MCE | # HY-130143 |
| tris (benzyltriazolylmethyl) amine (TBTA) | Sigma | #678937 |
| High Capacity NeutrAvidin agarose beads | Thermo | #29202 |
| protein A/G PLUS-agarose | Santa Cruz Biotechnology | #sc-2003 |
| 4% paraformaldehyde (PFA) | Solarbio | #IP9320 |
| lipofectamine™ 3000 transfection reagent | Thermo | # L3000015 |
| 0.25% trypsin-EDTA (m/V) | Thermo | #25200072 |
| Pronase | Roche | 10165921001 |
| Dextran Sulfate Sodium Salt (DSS) | MP Biomedicals | #0216011080 |
| Nrf2 antibody | Proteintech | #16396-1-AP |
| Keap1 antibody | Proteintech | #60027-1-Ig |
| Phospho-p62 (Ser349) antibody | Proteintech | #29503-1-AP |
| p62 antibody | Proteintech | #18420-1-AP |
| β-actin antibody | Proteintech | #66009-1-Ig |
| Occludin 1 antibody | Proteintech | #27260-1-AP |
| Claudin 1 antibody | Proteintech | #13050-1-AP |
| ZO-1 antibody | Proteintech | #21773-1-AP |
| HO-1 antibody | Abmart | #T55113 |
| GCLM antibody | Abmart | #T57202 |
| NQO1 antibody | Abmart | #T56710 |
| Ubiquitin antibody | Santa Cruz Biotechnology | #sc-8017 |
| IgG antibody | Santa Cruz | #sc-515946 |
| MAFG antibody | Abcam | #ab154318 |
| IL-1β antibody | Cell Signaling Technology | #12242 |
| CD86 antibody | HUABIO | #ET1606 |
| CD206 antibody | HUABIO | #ET1702-04 |
| Lamin B1 antibody | Proteintech | # 12987-1-AP |
| F4/80 antibody | Proteintech | # 29414-1-AP |
| iNOS antibody | Proteintech | # 22226-1-AP |
| TRIM21 antibody | Proteintech | #12108-1-AP |
| ZRANB1 Polyclonal antibody | Proteintech | #27050-1-AP |
| Lamp1 antibody | Proteintech | #32435-1-AP |
| IL-6 ELISA kit (Mouse) | Neobioscience | #EMC004.96 |
| CCL2/JE/MCP-1 ELISA kit (Mouse ) | Neobioscience | #EMC113.96 |
| IL-17/IL-17A ELISA kit (Mouse) | Neobioscience | #EMC008.96 |
| IL-10 ELISA kit (Mouse) | Neobioscience | #EMC005.96 |
| TNF-α ELISA kit (Mouse) | Neobioscience | #EMC102a.96 |
| IL-1β ELISA kit (Mouse) | Neobioscience | #EMC001b.96 |
| IL-23 ELISA kit (Mouse) | Neobioscience | #EMC114.96 |
| MPO ELISA kit (Mouse) | BOSTER | #EK0943 |
| IL-1β ELISA kit (Human) | Neobioscience | #EHC002b.96 |
| TNF-α ELISA kit (Human) | Neobioscience | #EHC103a.96 |
| IL-6 ELISA kit (Human) | Neobioscience | #EHC007.96 |
| Bovine Serum Albumin V (BSA) | Solarbio | #A8020 |
| Reactive Oxygen Species Assay Kit | Beyotime | #S0033S |
| CheKine™ Micro Lipid Peroxidation (MDA) Assay kit | Abbkine | #KTB1050 |
| CheKine™ Micro Superoxide Dismutases (SOD) Activity Assay Kit | Abbkine | # KTB1030 |
| CheKine™ Micro Reduced Glutathione (GSH) Assay Kit | Abbkine | # KTB1600 |
| CheKine™ Micro Catalase (CAT) Activity Assay Kit | Abbkine | # KTB1040 |
| IPKine™ HRP, Goat Anti-Mouse IgG LCS | Abbkine | #A25012 |
| IPKine™ HRP, Mouse Anti-Rabbit IgG LCS | Abbkine | #A25022 |
| CD3e Monoclonal Antibody | Thermo | #48-0031-80 |
| Invitrogen CD8a Monoclonal Antibody | Thermo | #11-0081-82 |
| CD4 Monoclonal Antibody | Thermo | #416-0042-80 |
| Invitrogen CD86 (B7-2) Monoclonal Antibody | Thermo | #12-0862-81 |
| Invitrogen CD206 (MMR)Monoclonal Antibody | Thermo | #17-2061-82 |
| Agarose | Solarbio | #A8201 |
| Sodium dodecylsulfate (SDS) | Solarbio | #S8010 |
| SPARKeasy Endofree Midi Plasmid Kit | Sparkjade Biotechnology | #AD0105 |
| Triton X-100 | Solarbio | #T8200 |
| NcmECL Ultra | NCM Biotech | #P10300 |
| Ad-mCherry-GFP-LC3B | Beyotime | # C3011 |

**Table S2.** Sequences of primers used for qPCR assay

| Genes | Forward | Reverse |
| --- | --- | --- |
| *IL-1β* (M) | ATGATGGCTTATTACAGTGGCAA | GTCGGAGATTCGTAGCTGGA |
| *IL-6* (M) | ACTCACCTCTTCAGAACGAATTG | CCATCTTTGGAAGGTTCAGGTTG |
| *CD206* (M) | CAGGTGTGGGCTCAGGTAGT | TGTGGTGAGCTGAAAGGTGA |
| *CD86* (M) | CTGCTCATCTATACACGGTTACC | GGAAACGTCGTACAGTTCTGTG |
| *IL-10* (M) | TCAAGGCGCATGTGAACTCC | GATGTCAAACTCACTCATGGCT |
| *Gapdh* (M) | AGATCCCTCCAAAATCAAGTGG | GGCAGAGATGATGACCCTTTT |
| *MCP-1* (M) | CAACTCTCACTGAAGCCAGCTC | TAGCTCTCCAGCCTACTCATTGG |
| *TNF-α* (M) | CCTCTCTCTAATCAGCCCTCTG | GAGGACCTGGGAGTAGATGAG |
| *iNOS* (M) | CCAAGCCCTCACCTACTTCC | CTCTGAGGGCTGACACAAGG |
| *Sqstm1* (M) | AGTGATGAGGAGCTGACAATGGCT | GCCAGCCAAAGTGTCCATGTTTCA |
| *Nqo1* (M) | GCCGAACACAAGAAGCTGGAAG | GGCAAATCCTGCTACGAGCACT |
| *Hmox1* (M) | GCTAGCCTGGTGCAAGATACT | TGGGGGCCAGTATTGCATTT |
| *Nrf2* (M) | GCCCACATTCCCAAACAAGAT | CCAGAGAGCTATTGAGGGACTG |
| *Usp8* (M) | AAAAATCCGATTACCCCAGC | GGCATCTTTGGTGTTCCAGT |
| *Zranb1* (M) | CAATGCTTGTGTGGGGGTTG | GCAAGCGTACTTCATCTGCG |
| *USP8* (H) | CCAAACTGAAGCGCTCCTAC | CTGGTCCAGAACCTCCAAAA |
| *ZRANB1*(H) | GGCTCTTCCTCAATGCTTGT | TGTCTCCTCCTGATGACTT |
| *IFN-γ* (H) | TCCAGGCATGCATACCGAAGACAA | ATGCTGCCAGGTTCAGACTGGTTA |
| *IL-1β* (H) | TTGACGGACCCCAAAAGAT | GAAGCTGGATGCTCTCATCTG |
| *IL-6* (H) | GTAGCCGCCCCACACAGA | CATGTCTCCTTTCTCAGGGCTG |
| *MCP-1* (H) | GAGGAAGGCCAGCCCAGCAC | TGGATGCTCCAGCCGGCAAC |
| *TNF-α* (H) | TGGCCCAGACCCTCACACTCAG | ACCCATCGGCTGGCACCACT |
| *IL-10* (H) | AAGGACCAGCTGGACAACAT | AGACACCTTTGTCTTGGAGCTTA |
| *Gapdh* (H) | CGGAGTCAACGGATTTGGTCGTAT | AGCCTTCTCCATGGTGGTGAAGAC |

**Table S3.** Genotyping Primers for Transgenic Mice

| primer set 1 | primer set 2 |
| --- | --- |
| (Positive for targeted gene knockout: 431 bp) | (The target gene knockout was negative: 440 bp) |
| F1: 5’-GAGCCTTAGTAGATGTCCCTTTACC-3’ | F1: 5’-GAGCCTTAGTAGATGTCCCTTTACC-3’ |
| R1: 5’-GCATATGCACATTCAAAACTCTCC-3’ | R2: 5’-GAGAGTCAAAGAGTCCATCCAGAAC-3’ |

**Table S4.** Label-free quantitative proteomic analysis

| **Protein names** | **Gene names** | **Score** | **Binding site positions** | **Fold of change（Drug/Control）** |
| --- | --- | --- | --- | --- |
| Peroxiredoxin-1 | Prdx1 | 323.31 | 173 | 1.64 |
| **Sequestosome-1** | **Sqstm1** | **323.31** | **26;113;219;291** | **3.25** |
| 60S acidic ribosomal protein P1 | Rplp1 | 126.21 | 9;61 | 0.57 |
| Proteasome assembly chaperone 1 | Psmg1 | 92.366 | 183 | 1.86 |
| Thioredoxin | Txn | 54.634 | 32;73 | 1.64 |
| Recombining binding protein suppressor of hairless | Rbpj | 32.338 | 339 | 1.52 |
| Scaffold attachment factor B1 | Safb | 27.522 | 225;303 | 1.88 |
| Protein KRI1 homolog | Kri1 | 19.722 | 554 | 0.00 |
| CLIP-associating protein 2 | Clasp2 | 15.484 | 227 | N/A |
| Transcriptional repressor CTCF | Ctcf | 14.777 | 155 | 0.00 |
| Tudor and KH domain-containing protein | Tdrkh | 14.432 | 109 | N/A |
| TBC1 domain family member 4 | Tbc1d4 | 13.737 | 909 | 0.00 |
| Tubulin beta-2B chain;Tubulin beta-2A chain | Tubb2b;Tubb2a | 10.191 | 127;129;239 | N/A |
| Nitric oxide-associated protein 1 | Noa1 | 6.6527 | 8;12 | 0.00 |
| Pre-mRNA-splicing regulator WTAP | Wtap | 5.2387 | 161 | N/A |
| SOSS complex subunit B1 | Nabp2 | 3.1701 | 81 | 0.00 |
| Zinc finger protein OZF | Znf146 | 2.2506 | 18 | 0.00 |
| Tubulin alpha-1A chain;Tubulin alpha-3 chain | Tuba1a;Tuba3a | 1.4782 | 20;25;295 | 0.00 |
| Thioredoxin-interacting protein | Txnip | 1.1407 | 36 | 0.00 |

**Figure S1. p62 knockdown mice exhibit an exacerbated inflammatory phenotype.** (A) Expression of p62 in normal colon tissue and colon cancer tissue (N: Normal; T: Tumor; n pairs=5). (B) Compared to normal colonic epithelial cells (NCM460), p62 protein expression was upregulated in colon cancer cells (HCT116, SW620, SW480, CT26) (n=3). (C) The increase of *SQSTM1* mRNA in colon cancer tissues (N: Normal; T: Tumor; n=5). (D) Expression levels of p62, p65, and iNOS proteins in murine liver and lung tissues (n=5). (E-F) mRNA levels of pro-inflammatory cytokines and *Sqstm1* in liver and lung tissues of acute inflammation models (n=5). (G) Representative H&E staining of lung tissues (n=5). (H) Protein expression levels of M1 marker (CD86) and M2 marker (CD206) in liver and lung tissues (n=5). (I-J) mRNA expression levels of M1 macrophage marker (*CD86*) and M2 markers (*CD206* and *CD163*) in liver and lung tissues (n=5). (K) Statistical quantification of colon length in UC mice (n=5). (L) Histopathological H&E scores of colon tissues in UC mice (n=5). (M) Quantification of pro-inflammatory cytokines in UC mouse colon tissues (n=5). (N) Protein expression of Occludin, Claudin-1, ZO-1, and p62 in colon tissues (n=5). (O) Representative immunohistochemical (IHC) staining for ZO-1, Occludin, and IL-1β in colon tissues (n=5). *^*^P* < 0.05, *^**^P* < 0.01 and *^***^P* < 0.001 vs. the WT- control, while *^#^P* < 0.05, *^##^P* < 0.01 and *^###^P* < 0.001 vs. the KO p62^-/-^- Control, while *^$^P* < 0.05, *^$$^P* < 0.01 and *^$$$^P* < 0.001 vs. the WT- LPS and WT- DSS (3%).

**Figure S2. p62 knockdown mice exhibit an exacerbated inflammatory phenotype.** (A) Representative immunofluorescence images of CD86 and F4/80 in colon tissue (n=5). (B-C) Expression of p62 and M1 markers (iNOS, IL-1β, CD86) in BMDMs and PMs (n=5). (D) mRNA expression of M1 macrophage markers (iNOS, IL-1β, CD86, IL-6, MCP-1, TNF-α) in PMs (n=5).  *^*^P* < 0.05, *^**^P* < 0.01 and *^***^P* < 0.001 vs. the WT- control, while *^#^P* < 0.05, *^##^P* < 0.01 and *^###^P* < 0.001 vs. the KO p62^-/-^- Control, while *^$^P* < 0.05, *^$$^P* < 0.01 and *^$$$^P* < 0.001 vs. the WT- LPS and WT- DSS (3%).

**Figure S3. p62 enhancement by the natural product-derived activator αMγB attenuates UC.** (A) Immunofluorescence staining for p62 (n=3). (B) Protein expression of p62 in RAW264.7 cells (n=3). (C) Validation of p62 knockdown. Transfection with 60 pmol of si-*Sqstm1* achieved the highest silencing efficiency at both mRNA and protein levels (n=3). (D) Protein expression of iNOS, IL-1β, CD206 and CD86 after p62 knockdown (n=3). (E) Cytokine production after p62 knockdown (n=3). (F) p62 silencing reverses the effects of αMγB on cytokine mRNA levels, suppressing pro-inflammatory and enhancing anti-inflammatory expression (n=3). (G) Cytotoxicity of αMγB toward NCM460 cells (MTT assay) (n=6). (H) Protein expression of p62 in NCM460 cells (n=3). (I) αMγB reduces pro-inflammatory cytokine mRNA and elevates *IL-10* mRNA in NCM460 cells (n=3). (J) Inhibition of THP-1 cell adhesion to NCM460 cells by αMγB (n=3). (K-M) In macrophage-epithelial cocultures, αMγB blocks TNF-α-induced macrophage inflammatory responses mediated by epithelial cells (n=3). (N) Quantification of colon length in UC mice (n=5). (O) Colon H&E score in UC mice (n=5). (P) Expression of intestinal epithelial tight junction proteins, including ZO-1, Occludin 1 and Claudin-1 in colon tissue (n=5). (Q-X) Levels of SOD, MDA, GSH, and CAT in murine serum and colon tissue (n=5). (Y-Z) αMγB-promoted expression of tight junction proteins (ZO-1, Occludin 1, Claudin-1) in colon tissue was reversed by ML385 (n=5).  *^*^P* < 0.05, *^**^P* < 0.01 and *^***^P* < 0.001 vs. the Control, the si-NC, while *^#^P* < 0.05, *^##^P* < 0.01 and *^###^P* < 0.001 vs. the si-NC+ LPS (200 ng/mL), the TNF-α (20 ng/mL) and the DSS (3%), while *^$^P* < 0.05, *^$$^P* < 0.01 and *^$$$^P* < 0.001 vs. the si-NC+ LPS+αMγB, the DSS+ αMγB, while *^&^P* < 0.05, *^&&^P* < 0.01 and *^&&&^P* < 0.001 vs. the Costunolide.

**Figure S4. p62 enhancement by the natural product-derived activator αMγB attenuates UC.** (A-B) Immunohistochemical analysis of tight junction proteins (ZO-1 and Occludin 1) as well as pro-inflammatory IL-1β in colon tissue (Scale bar = 50 μm; n=5). (C-H) αMγB suppressed pro-inflammatory cytokines while elevating anti-inflammatory cytokines in the serum and the colon tissue of UC mice, at both protein and mRNA levels. This effect was reversed by ML385 (n=5). (I-J) Immunohistochemical analysis of macrophage markers for M1 (CD86) and M2 (CD206) polarization in colon tissue (Scale bar = 50 μm; n=5). (K-L) Immunofluorescence analysis for macrophages and their polarization states in colon tissue, using markers F4/80 (pan), CD86 (M1), and CD206 (M2) (Scale bar = 50 μm; n=5). *^*^P* < 0.05, *^**^P* < 0.01 and *^***^P* < 0.001 vs. the Control, while *^#^P* < 0.05, *^##^P* < 0.01 and *^###^P* < 0.001 vs. the DSS (3%), while *^$^P* < 0.05, *^$$^P* < 0.01 and *^$$$^P* < 0.001 vs. the DSS+ αMγB, while *^&^P* < 0.05, *^&&^P* < 0.01 and *^&&&^P* < 0.001 vs. the Costunolide.

**Figure S5.** **p62 enhancement by the natural product-derived activator αMγB attenuates UC.** (A-B) αMγB significantly inhibited M1 polarization (CD86) while promoting M2 polarization (CD206) in colonic tissues, an effect reversed by the Nrf2 inhibitor ML385 (n=5). (C) Gating strategy for the identification of CD3e^+^CD4^+^CD8α^+^ T cell subsets. (D) αMγB effectively restored the CD4^+^/CD8α^+^ ratio in the spleen and mesenteric tissues of UC mice, which was subsequently reversed by ML385 (n=5). (E) Rarefaction curves of the Sobs index at the ASV level (n=5). (F-G) Analysis of gut microbiota α-diversity using Ace and Shannon indices at the Amplicon Sequence Variant (ASV) level (n=5). (H) Principal coordinates analysis (PCoA) plots revealing structural divergence in gut microbiota communities (n=5). (I) LEfSe analysis identifying differentially abundant bacterial taxa from phylum to genus levels (n=5). (J-M) αMγB significantly upregulated p62 protein expression in the colons of UC mice, and this effect was reversed by ML385 (n=5). (N) Schematic of establishing the p62-knockout mice model of UC. (O) Quantification of colon length in UC mice (n=5). (P) Colon H&E score in UC mice (n=5). (Q-R) In DSS-induced UC using p62-knockout mice, αMγB failed to restore colonic tight junction proteins (Occludin 1, Claudin 1, ZO-1) (n=5). *^*^P* < 0.05, *^**^P* < 0.01 and *^***^P* < 0.001 vs. the Control, while *^#^P* < 0.05, *^##^P* < 0.01 and *^###^P* < 0.001 vs. the DSS (3%), while *^$^P* < 0.05, *^$$^P* < 0.01 and *^$$$^P* < 0.001 vs. the DSS+ αMγB, while *^&^P* < 0.05, *^&&^P* < 0.01 and *^&&&^P* < 0.001 vs. the Costunolide.

**Figure S6. p62 enhancement by the natural product-derived activator αMγB attenuates UC.** (A) Histopathological evaluation of major organs following αMγB administration. Representative H&E-stained sections of the heart, liver, spleen, lung, and kidney from healthy mice treated with PBS or αMγB (10 mg/kg/day, i.p.) for 14 consecutive days. No apparent histopathological abnormalities were observed in the αMγB-treated group compared with the control group (Scale bar = 100 μm; n=5).

**Figure S7. αMγB activates Nrf2 through p62-mediated autophagic-lysosomal degradation of Keap1.** (A) RNA-seq analysis reveals αMγB-mediated upregulation of Nrf2-regulated antioxidant genes (*Hmox1, Nqo1, Gclm, etc.*). (B-I) αMγB significantly upregulates Nrf2 and its downstream proteins (NQO1, GCL, HO-1) and promotes Keap1 degradation in RAW264.7 and NCM460 cells (n=3). (J-K) αMγB significantly upregulated the protein levels of Nrf2 and its downstream targets (NQO1, HO-1, and GCL) while promoting Keap1 degradation in the colon tissues of UC mice, an effect that was reversed by the Nrf2 inhibitor ML385 (n=5). (L) αMγB enhances nuclear translocation and expression of Nrf2 (n=3). (M) The ability of αMγB to suppress M1 markers (CD86, iNOS, IL-1β) and induce the M2 marker CD206 was reversed by ML385 (n=3). (N) αMγB's suppression of LPS-induced NO production is abrogated by ML385 (n=3). (O-P) αMγB enhances p62-Keap1 interactions in RAW264.7 and NCM460 cells (n=3). (Q) p62 knockdown leads to inhibition of the p62-Keap1-Nrf2 pathway (n=3). (R-S) p62 silencing abolished αMγB-mediated upregulation of Nrf2-target proteins (NQO1, HO-1, GCL), as well as Keap1 degradation (n=3). *^*^P* < 0.05, *^**^P* < 0.01 and *^***^P* < 0.001 vs. the Control or the si-NC, while *^#^P* < 0.05, *^##^P* < 0.01 and *^###^P* < 0.001 vs. the LPS or the si-NC+ LPS, while *^$^P* < 0.05, *^$$^P* < 0.01 and *^$$$^P* < 0.001 vs. the LPS+ αMγB or the si-NC+ LPS+ αMγB.

**Figure S8. αMγB activates Nrf2 through p62-mediated autophagic-lysosomal degradation of Keap1.** (A-B) p62 silencing abolished αMγB-mediated upregulation of Nrf2-target proteins (NQO1, HO-1, GCL), as well as Keap1 degradation (n=3). (C-E) The activating effect of αMγB on the p62-Keap1-Nrf2 signaling pathway was reversed by ML385 (n=3). (F-H) αMγB promotes the expression of phosphorylated p62 (P-p62) in a time- and dose-dependent manner (n=3). *^*^P* < 0.05, *^**^P* < 0.01 and *^***^P* < 0.001 vs. the Control, while *^#^P* < 0.05, *^##^P* < 0.01 and *^###^P* < 0.001 vs. the LPS, while *^$^P* < 0.05, *^$$^P* < 0.01 and *^$$$^P* < 0.001 vs. the LPS+ αMγB.

**Figure S9. αMγB stabilized p62 expression by targeting and blocking its ubiquitination.** (A) p62 expression in response to treatment with MG132, CQ, and αMγB, either alone or in combination (n=3). (B) αMγB enhances disruption of p62 ubiquitination in NCM460 cells (n=3). (C) Identification of p62-associated deubiquitinating enzymes (DUBs) using the UbiBrowser database (http://ubibrowser.bio-it.cn/). (D-E) αMγB had no significant effect on the mRNA levels of ZRANB1, USP15, and USP20 (human), nor Zranb1 and Usp8 (mouse) (n=3). (F) No significant changes in the protein levels of ZRANB1 were observed in RAW264.7 and NCM460 cells following αMγB treatment (n=3). (G-H) The interaction between p62 and ZRANB1 remained unchanged following αMγB treatment in RAW264.7 and NCM460 cells (n=3). (I-K) NAC reverses αMγB's suppression of NO and M1 markers (CD86, iNOS, IL-1β, TNF-α, IL-6, MCP-1) and induction of M2 markers (CD206, IL-10) (n=3). (L-O) NAC reversed the αMγB-induced upregulation of p62, Nrf2, and downstream proteins, as well as Keap1 degradation (n=3). *^*^P* < 0.05, *^**^P* < 0.01 and *^***^P* < 0.001 vs. the Control, while *^#^P* < 0.05, *^##^P* < 0.01 and *^###^P* < 0.001 vs. the LPS, while *^$^P* < 0.05, *^$$^P* < 0.01 and *^$$$^P* < 0.001 vs. the LPS+ αMγB.

**Figure S10. αMγB prevents exposure of 297-E3-linked ubiquitin sites by alkylation of cysteine 291.** (A-L) Molecular docking simulations demonstrate covalent binding of αMγB to four cysteine residues on p62: C26, C113, C219, and C291. (M-N) Mutant constructs targeting p62 (WT), p62 (C26S), p62 (C113S), p62 (C219S), and p62 (C291S) sites all show detectable expression in transfected RAW264.7 cells (n=3). (O) Quantification of Nrf2 fluorescence intensity (n=3). (P-Q) p62 (C291S) mutation partially blocks αMγB-induced activation of the p62-Keap1-Nrf2 signaling pathway (n=3).  *^*^P* < 0.05, *^**^P* < 0.01 and *^***^P* < 0.001 vs. the Vector, while *^#^P* < 0.05, *^##^P* < 0.01 and *^###^P* < 0.001 vs. the Vector+ LPS, while *^$^P* < 0.05, *^$$^P* < 0.01 and *^$$$^P* < 0.001 vs. the p62 (WT)+ LPS + αMγB.

**Figure S11. αMγB fails to inhibit macrophage M1 polarization and alleviate UC in *Sqstm1*^C291S^ mutant mice.** (A) Colon length across experimental groups (n=5). (B) Histopathological scoring of colon tissue sections (n=5). (C) Reconstitution with *Sqstm1*^WT^ rescues αMγB's ability to suppress pro-inflammatory cytokines (IL-6, IL-1β, TNF-α, MCP-1, IL-17, IL-23) and MPO levels while promoting IL-10 expression in colon tissues of DSS-induced UC *Sqstm1*^-/-^ mice. This rescue is abrogated in mice reconstituted with the *Sqstm1*^C291S^ mutant (n=5). (D) Expression of intestinal epithelial tight junction proteins, including ZO-1, Claudin 1 and Occludin, in colon tissue (n=5). (E) Expression of macrophage polarization markers in BMDMs, including M1 (CD86, iNOS, IL-1β) and M2 (CD206) proteins (n=5). (F) Immunofluorescence analysis for macrophages and their polarization states in colon tissue, using markers F4/80 (pan), CD86 (M1), and CD206 (M2) (n=5). (G-J) Reconstitution of *Sqstm1*^WT^ fully restored the regulatory effects of αMγB on the polarization imbalance in macrophages (BMDMs and PMs), whereas the *Sqstm1*^C291S^ mutant failed to rescue these effects (n=5). *^*^P* < 0.05, *^**^P* < 0.01 and *^***^P* < 0.001 vs. the AAV-9- *Sqstm1*^WT^- Control, while *^#^P* < 0.05, *^##^P* < 0.01 and *^###^P* < 0.001 vs. the AAV-9- *Sqstm1*^WT^- DSS (3%), while *^$^P* < 0.05, *^$$^P* < 0.01 and *^$$$^P* < 0.001 vs. the AAV-9- *Sqstm1*^C291S^- Control.

**Figure S12. αMγB fails to inhibit macrophage M1 polarization and alleviate UC in *Sqstm1*^C291S^ mutant mice.** (A-C) Reconstitution of *Sqstm1*^WT^ fully restored the activation of the p62-Keap1-Nrf2 pathway by αMγB, whereas overexpression of the *Sqstm1*^C291S^ mutant failed to rescue this activation (n=5). *^*^P* < 0.05, *^**^P* < 0.01 and *^***^P* < 0.001 vs. the AAV-9- *Sqstm1*^WT^ - Control, while *^#^P* < 0.05, *^##^P* < 0.01 and *^###^P* < 0.001 vs. the AAV-9- *Sqstm1*^WT^ - DSS (3%), while *^$^P* < 0.05, *^$$^P* < 0.01 and *^$$$^P* < 0.001 vs. the AAV-9- *Sqstm1*^C291S^ - Control.
